# Somatic *Miwi2* modulates mitochondrial function in airway multiciliated cells and exacerbates influenza pathogenesis

**DOI:** 10.1016/j.isci.2025.112291

**Published:** 2025-03-25

**Authors:** Jhonatan Henao Vasquez, Jin Yuan, Chi Jing Leow, Erin Crossey, Fengzhi Shao, Senegal Carty, Viviana A. Dominguez, Ming Lo, Joseph P. Mizgerd, Jessica L. Fetterman, Nelson C. Lau, Alan Fine, Matthew R. Jones

**Affiliations:** 1 The Pulmonary Center, Boston University Chobanian & Avedisian School of Medicine, Boston, MA 02118, USA; 2 Department of Medicine, Boston University Chobanian & Avedisian School of Medicine, Boston, MA 02118, USA; 3 Department of Biochemistry and Cell Biology, Boston University Chobanian & Avedisian School of Medicine, Boston, MA 02118, USA; 4 National Emerging Infectious Diseases Laboratories, Comparative Pathology Laboratory, Boston University Chobanian & Avedisian School of Medicine, Boston, MA 02118, USA; 5 Department of Pathology and Laboratory Medicine, Boston University Chobanian & Avedisian School of Medicine, Boston, MA 02118, USA; 6 Department of Virology, Immunology & Microbiology, Boston University Chobanian & Avedisian School of Medicine, Boston, MA 02118, USA; 7 Boston University Genome Science Institute, Boston University Chobanian & Avedisian School of Medicine, Boston, MA 02118, USA

**Keywords:** Biochemistry, Cell biology, Functional aspects of cell biology

## Abstract

MIWI2, a P element-induced wimpy testes (PIWI) argonaute protein known for suppressing retrotransposons during male gonadogenesis, has an unexplored role in mammalian somatic cells. We identify MIWI2 multiciliated (M2MC) cells as a rare subset of airway multiciliated cells and investigate MIWI2’s function in antiviral host defense. We analyzed transcriptomes from *Miwi2* heterozygous (*Miwi2*^+/tom^) and deficient (*Miwi2*^tom/tom^) mice following influenza A infection. During infection, *Miwi2* deficiency was associated with reduced mitochondrial and ribosomal gene expression in M2MC cells, increased mitochondrial reactive oxygen species (ROS) production and ADP/ATP ratios in multiciliated cells, and enhanced viral clearance and recovery. Additionally, *Miwi2*-expressing cells exhibited reduced levels of small RNAs derived from nuclear mitochondrial DNA. These findings reveal a previously unrecognized role for *Miwi2* in regulating small non-coding RNAs and mitochondrial oxidant production in somatic cells, indicating a function beyond its established germline activities. Our study identifies *Miwi2*/*Piwil4* as a potential factor influencing susceptibility to severe respiratory infections.

## Highlights

• *Miwi2* upregulates select mitochondrial genes in M2MC cells during IAV infection
• *Miwi2* reduces intracellular ROS and ADP/ATP ratios in multiciliated cells
• NuMT derived small RNAs are decreased in *Miwi2* expressing cells
• *Miwi2* deficient mice experience decreased viral burden from IAV

## Graphical abstract

**Figure:**
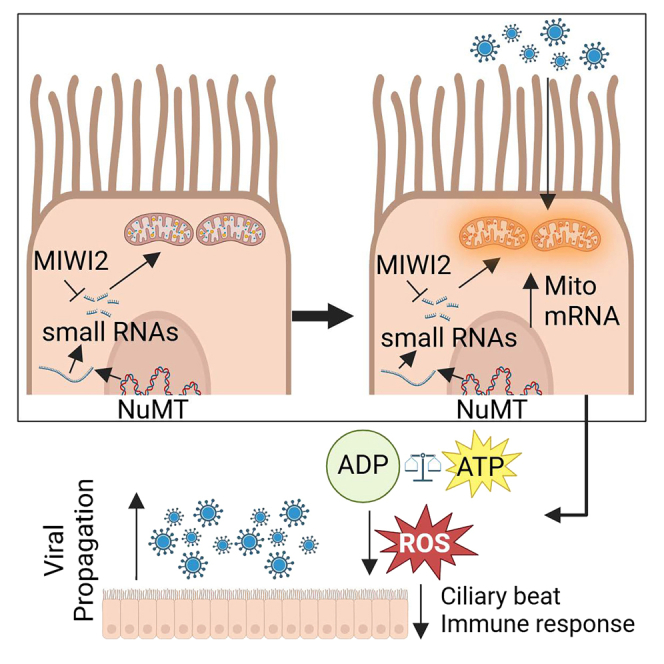

## Introduction

Respiratory infections are among the leading causes of morbidity and mortality, with influenza alone causing approximately 35–65 million infections and 25,000–72,000 deaths in the US during the 2023–2024 season.^1^ Despite the availability of vaccines and antiviral drugs, their effectiveness varies, especially among high-risk patients^2^^,^^3^; thus underscoring a critical need to further our understanding of the host response during an influenza infection to develop more effective therapeutic strategies.

Host defense against respiratory viruses is reliant on an orchestrated release of cytokine and chemokine signals originating from infected cells of the pulmonary epithelium, which line the respiratory tract from the trachea to alveoli.^4^^,^^5^^,^^6^ These signals initiate and modulate host immune responses; recruit neutrophils, T cells, and other immune cells to the site of infection; and activate macrophages.^7^^,^^8^^,^^9^^,^^10^ The influenza A virus (IAV) binds to sialic acid glycoprotein receptors found on the surface of airway epithelial cells, which include multiple cell types such as type I and II pneumocytes, basal cells, secretory club cells, and multiciliated cells.^9^^,^^11^^,^^12^^,^^13^ Multiciliated cells, which are most abundant in the conducting airways, primarily function to maintain the mucociliary escalator over the luminal surface.^14^^,^^15^^,^^16^^,^^17^ Like other epithelial cell types, multiciliated cells are heterogeneous, as demarcated by differential gene expression.^18^^,^^19^^,^^20^^,^^21^^,^^22^^,^^23^ This heterogeneity confers multiciliated cells with a diverse range of functions that extend beyond just ciliary beating, enabling them to potentially play broader and more versatile roles in immune responses.^18^^,^^19^^,^^21^

Previously, we identified the MIWI2 multiciliated (M2MC) cell, a rare subpopulation of multiciliated cells that exclusively express MIWI2, the mouse ortholog of the human P element-induced wimpy testes like 4 (PIWIL4) argonaute family protein. Similarly, we found that PIWIL4 distinguishes a rare subpopulation of multiciliated cells in the human airway.^23^ Most abundantly expressed in male germline stem cells, MIWI2 forms a repressive complex with PIWI-interacting RNAs (piRNAs) to suppress retrotransposons (RTs) that are abundant transposable elements in mammalian genomes.^24^^,^^25^^,^^26^^,^^27^^,^^28^^,^^29^ This complex inhibits many major classes of RTs such as the long terminal repeat (LTR) and non-LTR RTs.^30^^,^^31^^,^^32^ Through complementary base-pairing with piRNAs, the RT RNA is cleaved by another PIWI protein, MILI, which can then be processed into additional piRNAs through ping pong amplification.^25^^,^^33^^,^^34^^,^^35^ Effector piRNAs loaded onto MIWI2 translocate to the nucleus, identifies nascent RT RNA, and recruits silencing factors for methylation of the promoter regions.^32^^,^^36^ This suppressive function of MIWI2 is essential in the male germline, ensuring stability of the genome. In cases of MIWI2 deficiency or dysfunction, meiotic arrest is induced in spermatogonia leading to male infertility.^37^

Outside of the germline, the role of PIWI-piRNA pathways has remained largely unexplored although some reports hint at regulatory functions in somatic cells. For example, PIWI-piRNA interactions have been examined in the pathogenesis of Alzheimer’s disease, multiple sclerosis, cardiovascular disease, and cancer.^38^^,^^39^^,^^40^^,^^41^^,^^42^ Additionally, PIWI-piRNA pathways have been investigated in viral infections such as in Respiratory Syncytial Virus (RSV), Human immunodeficiency virus (HIV), SARS-CoV-2, and Human Papillomavirus (HPV).^43^^,^^44^^,^^45^^,^^46^^,^^47^ In the context of respiratory infections, these mechanisms could influence how host cells respond to viral invasion, potentially impacting the severity and progression of disease. By investigating MIWI2’s role in viral infection, we may gain further insights into broader mechanisms of host-pathogen interactions and reveal potential therapeutic targets for not only influenza but for other respiratory viruses as well.

In this report, we elucidate the role of *Miwi2* in lung airway multiciliated cells on influenza pathogenesis and the host response to infection. To further investigate how *Miwi2* affects the host defense, we used a *Miwi2-tdTomato* knock-in reporter mouse model to isolate M2MC and nonMIWI2 multiciliated (nonM2MC) cells for RNA sequencing analysis of both long and small RNAs. The transcriptional profiles provided evidence of *Miwi2*-dependent expression of nuclear mitochondrial (NuMT) derived small RNAs and select mitochondrial mRNAs. Moreover, we observed a *Miwi2*-dependent modulation of mitochondrial transcripts that was associated with increased reactive oxygen species (ROS) levels and ADP/ATP ratios in multiciliated cells. Lastly, mice deficient in *Miwi2* exhibited improved outcomes when infected with IAV suggesting *Miwi2* as a key host susceptibility factor. Our work advances the understanding of *Miwi2* in somatic cells and highlights its pivotal role to modulate a class of NuMT-derived small RNAs and mitochondrial ROS function during infection.

## Results

### *Miwi2* heterozygous and deficient experimental models

**Figure 1:**
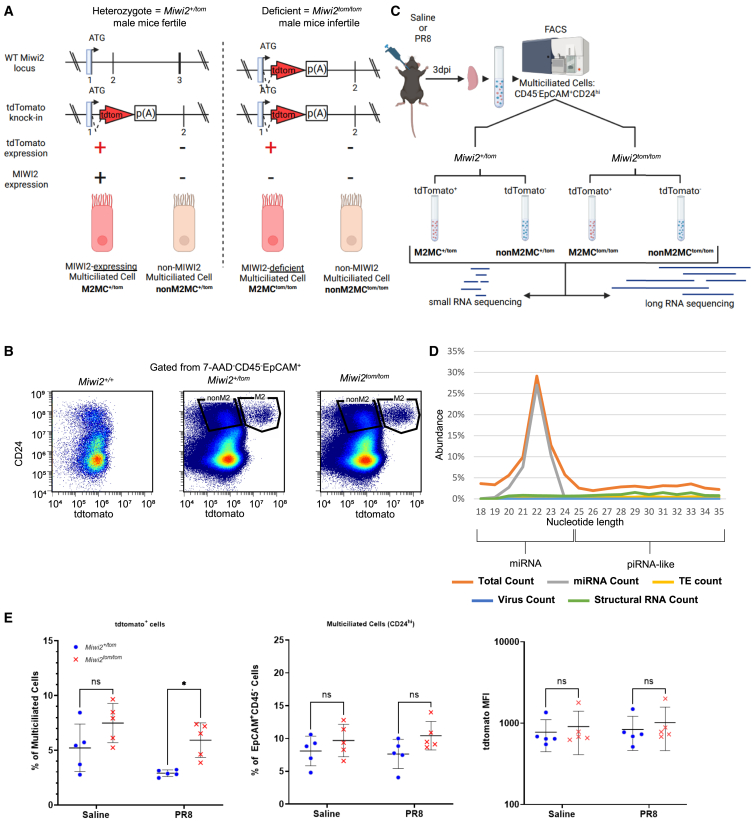
Schematic of *Miwi2-tdTomato* mice and experimental strategy (A) A *tdTomato* expression cassette is inserted into the first exon of *Miwi2*, inactivating the allele. Single allele knock-in (*Miwi2*^*+/tom*^) allows male mice to still be fertile, consistent with *Miwi2* haplosufficiency. Double allele knock-in (*Miwi2*^*tom/tom*^) induces infertility in males, consistent with lack of *Miwi2* activity. *p(A): poly(A) tail*. (B) Using multiciliated cell markers (CD45^−^EpCAM^+^CD24^hi^), the M2MC subset can be identified via flow cytometry, even in *Miwi2* deficient mice. (C) *Miwi2*^*+/tom*^ and *Miwi2*^*tom/tom*^ were intratracheally instilled with saline or PR8 3 days post infection into the left lobes (*n* = 4, 5–6 left lobes collected per *n*). Single cell suspensions were generated through an epithelial enrichment protocol. M2MC and nonM2MC cells were sorted based on multiciliated cell markers and tdTomato expression. Small and long RNAs were sequenced for each sample. (D) For small RNA sequencing, length distribution plots were used to filter piRNA-like small RNAs that were 24–35 nucleotides in length. (E) Flow cytometry quantification of tdTomato^+^ cells as a percentage of total multiciliated cells (*two-way ANOVA followed by Sidak’s test*). TdTomato mean fluorescence intensity (MFI) was also measured (*n* = 5). Total multiciliated cells (CD24^hi^) were quantified as a percentage of total epithelial cells (EpCAM^+^CD45^−^). Error bars represent mean ± standard deviation. *∗p < 0.05.*

A *Miwi2-tdTomato* knock-in reporter mouse model, previously described, was used to generate *Miwi2* heterozygous (*Miwi2*^*+/tom*^) and deficient (*Miwi2*^*tom/tom*^) mice (Figure 1A).^23^^,^^48^ Combining established surface markers (CD45^−^EpCAM^+^CD24^hi^) with tdTomato expression enabled the separation of M2MC and nonM2MC cells via flow cytometry, including cells from *Miwi2*^*tom/tom*^ mice (Figure 1B).^23^^,^^49^ This suggests that while M2MC cells are identified by the expression of *Miwi2*, *Miwi2* itself is not necessary for the development and presence of this subset.

To examine transcriptomic changes in both small and long RNA, M2MC and nonM2MC cells were isolated from *Miwi2*^*+/tom*^ and *Miwi2*^*tom/tom*^ mice intratracheally instilled with saline or mouse-adapted influenza A/Puerto Rico/8/34 (PR8) 3 days post infection (dpi) (Figure 1C).^50^ Small and total long RNA libraries were then constructed for each sample. Among all samples that passed quality control, miRNAs were the predominant class of small RNAs (Figures 1D and S1). Read length distributions were filtered between 24 and 35 nucleotides to analyze the piRNA-like small RNAs in the multi-species small RNA genomics (MSRG) pipeline.^51^^,^^52^ MicroRNAs (miRNAs) were separately analyzed through the COMPSRA pipeline.^53^ We observed more M2MC cells in *Miwi2*^*tom/tom*^ mice compared to *Miwi2*^*+/tom*^ mice during PR8 infection without an increase in tdTomato detection or significant changes in the overall amount of multiciliated cells (Figure 1E).

### Somatic retrotransposon expression induced by IAV, but is independent of *Miwi2*

During spermatogenesis, MIWI2 regulates the expression of two major classes of retrotransposons: long-terminal repeats (LTRs) and non-LTRs. Endogenous retroviruses (ERVs) are a commonly expressed family of LTRs that share a genetic structure and mechanism similar to exogenous retroviruses.^54^^,^^55^ The most common mammalian non-LTR family is LINE-1 that contains 2 open reading frame proteins that function in the transcription and integration of the RT into the genome.^30^^,^^56^^,^^57^ In MIWI2 deficient testes, these RTs become unregulated, induce meiotic arrest, and ultimately infertility.^58^^,^^59^ While MIWI2 represses these sites, recent studies suggest that viral infection may derepress them and stimulate antiviral immunity through proximal *cis*-acting enhancement of nearby antiviral genes or through activation of Pathogen-associated molecular pattern (PAMP) receptors inducing interferon (IFN) activation.^60^^,^^61^^,^^62^^,^^63^^,^^64^ Therefore, expression of RTs was examined in the long and small RNA sequencing dataset derived from the sorted multiciliated cells.

**Figure 2:**
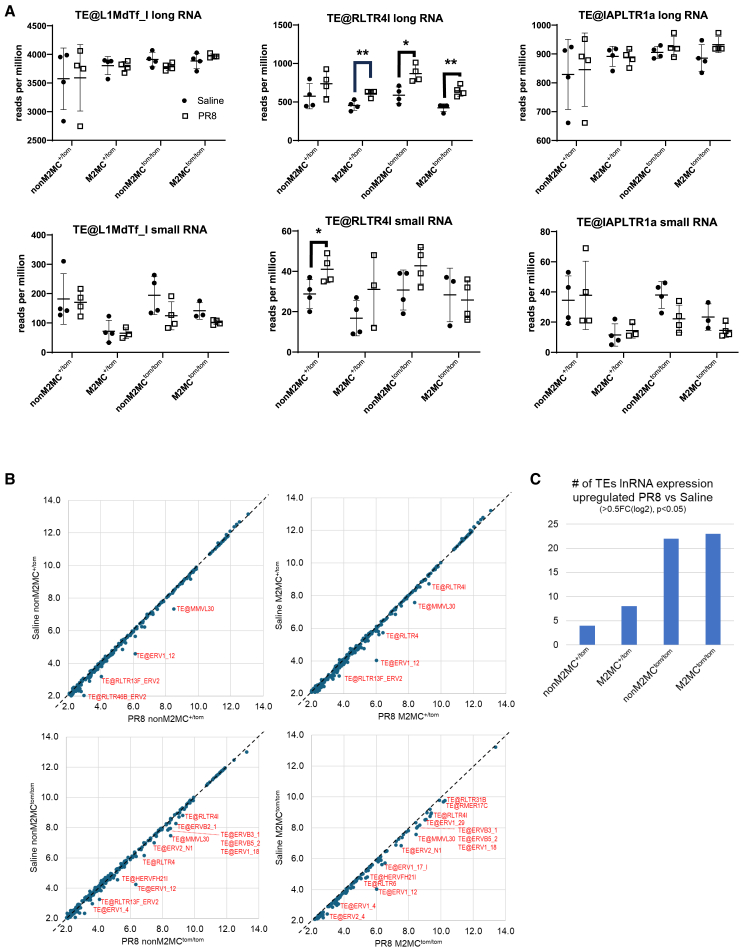
Retrotransposon expression is independent of *Miwi2* but is upregulated during PR8 infection (A) Long and small RNAs of the top 3 expressed RT subfamilies (*Student’s t test*). (B) Comparisons of matched cell types and genotypes between saline and PR8 treatments showed a set of mouse RTs whose expression was repeatedly enhanced, as marked with red labels for the RT type (*p < 0.05 by Student’s t test*). (C) Number of significantly upregulated RTs induced by PR8 infection for each represented group. Error bars represent mean ± standard deviation. *∗p < 0.05, ∗∗p < 0.005*.

When aligning total long and piRNA-like small RNAs to RTs of the mouse genome using the MSRG pipeline,^52^ no *Miwi2*-dependent changes were observed in the long or small RNAs of the highest expressed RTs (Figure 2A). Additional analysis of detected RTs also showed no genotype or cell type dependent difference (Figure S2A). Notably, a PR8-induced upregulation in several RT subfamilies were observed across all groups, predominantly ERV subfamilies (Figure 2B). In particular, more RT subfamilies were increased in both M2MC and nonM2MC cells of *Miwi2*^*tom/tom*^ mice, but only during PR8 infection (Figure 2C). Protein expression of the open reading frame 1 protein (ORF1p) derived from LINE-1 was analyzed in whole lung and sorted epithelial cells of saline and PR8 treated wildtype (*Miwi2*^+/+^) and *Miwi2*^*tom/tom*^ mice by western blot analyses.^65^^,^^66^ No changes in ORF1p were observed in the whole lung and it was undetectable in sorted epithelial cells (Figures S2B, S3A, and S3B). Taken together, these data indicate that IAV induces RT RNA expression in all multiciliated cells. However, it does not appear *Miwi2* alone impacts the steady-state expression of RT long and small RNAs in multiciliated cells.

### Influenza A viral-derived small RNAs are expressed independent of *Miwi2*

**Figure 3:**
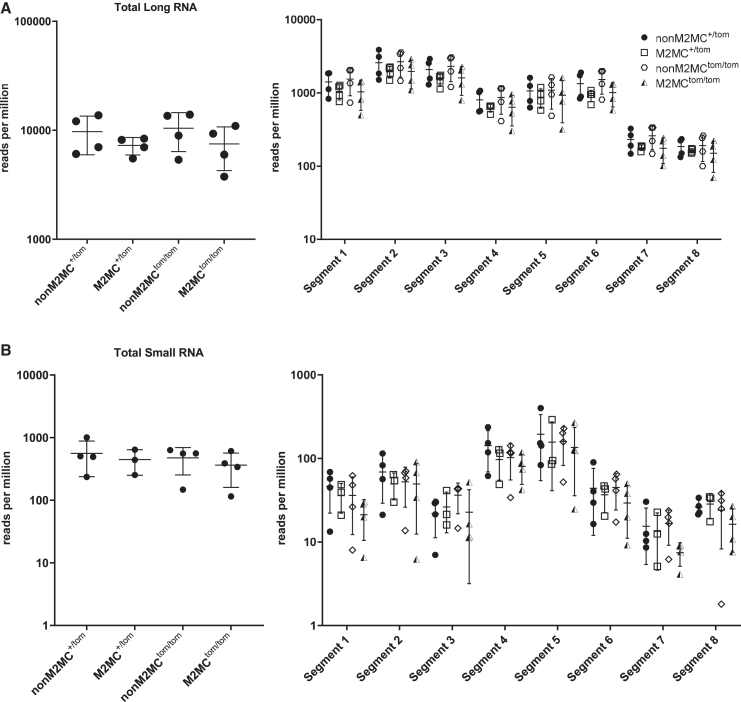
*Miwi2* does not alter PR8-derived RNAs during early infection Total (A) long and (B) small RNA reads that aligned to the PR8 genome. Reads were also separately quantified for each of the eight segments of the viral genome. Error bars represent mean ± standard deviation.

In insects, somatic cells might utilize the PIWI-piRNA pathway as a host defense mechanism against viral infections. Insects can generate piRNAs from the viral genome, and this processing of the viral RNA suppresses virion production and replication.^67^^,^^68^ We investigated whether airway multiciliated cells—among the first infected cells of the upper respiratory tract during influenza—may similarly generate viral-derived piRNAs, which could regulate RNA transcription and replication.^69^ We leveraged the MSRG pipeline to align long and small RNA reads to all 8 segments of the mouse-adapted PR8 genome. We observed no significant changes in total long and small RNA counts among the PR8 samples (Figures 3A and 3B). This was also true when comparing each segment separately. In addition, the coverage plots of long and small RNA reads along the viral genome were unchanged in all samples (Figures S4 and S5). While there may be small RNAs derived from the PR8 genome, their generation is independent of *Miwi2* and does not affect overall viral RNA transcription at early stages of infection.

### *Miwi2* modulates ribosomal and mitochondrial gene expression during acute infection

**Figure 4:**
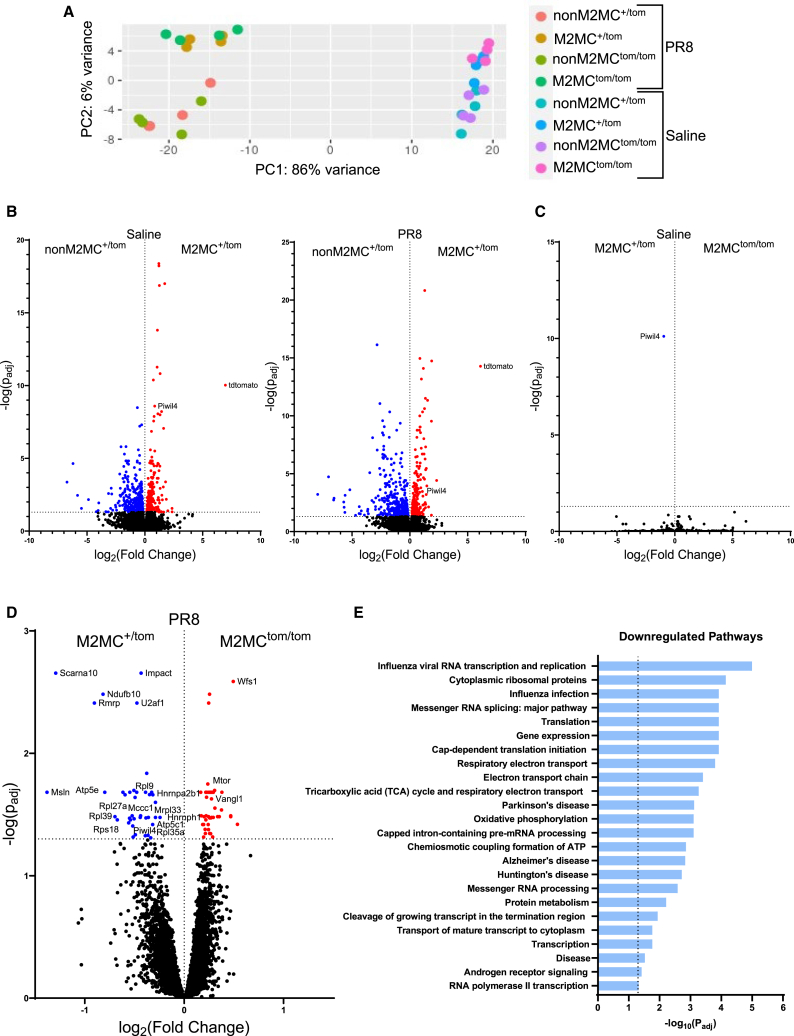
*Miwi2* deficiency is associated with a downregulation of mitochondrial and ribosomal genes in M2MC cells during early PR8 infection (A) Principal-component analyses of all long RNA samples. (B) Volcano plots comparing differentially expressed genes between nonM2MC^+/tom^ (blue) to M2MC^+/tom^ (red) cells under saline and PR8 conditions. (C and D) Volcano plots showing differentially expressed gene between M2MC^+/tom^ (blue) and M2MC^tom/tom^ (red) under (C) saline and (D) PR8 conditions. *FDR < 0.05*. (E) Pathway analysis on downregulated genes associated with *Miwi2* deficiency in PR8 infected M2MC cells. *Dotted line: FDR < 0.05*.

A third possible avenue of MIWI2’s function in airway multiciliated cells could be the regulation of host genes. Principal-component analyses of the total RNA shows M2MC cells cluster separately from nonM2MC cells, independent of *Miwi2* expression. The separation is more apparent during PR8 infection (Figure 4A). As anticipated, *Stat1*, IFN-stimulated, and IAV genes were upregulated when comparing PR8 to saline treated samples (Figure S6). Differential expression analysis shows that M2MC cells contain *Miwi2* and *tdTomato* transcripts, and are transcriptomically distinct from nonM2MC cells (Figure 4B) as seen with our previous studies.^23^ This also holds true when examining differentially expressed miRNAs (Figure S7A).

Strikingly, there were no *Miwi2* dependent gene changes in M2MC cells under saline conditions besides the *Miwi2* transcript itself (Figure 4C). During PR8 infection, however, select mitochondrial and ribosomal genes were downregulated, which was associated with *Miwi2* deficiency (Figure 4D). No *Miwi2*-dependent changes were observed in nonM2MC cells (Figure S7B). Pathway enrichment analysis of the downregulated genes in association with *Miwi2* deficiency indicated pathways involved in influenza viral RNA transcription, mitochondrial, and ribosomal function (Figure 4E; Table S1). Pathway enrichment analysis was also performed on the upregulated genes, but no significant findings were found.

**Figure 5:**
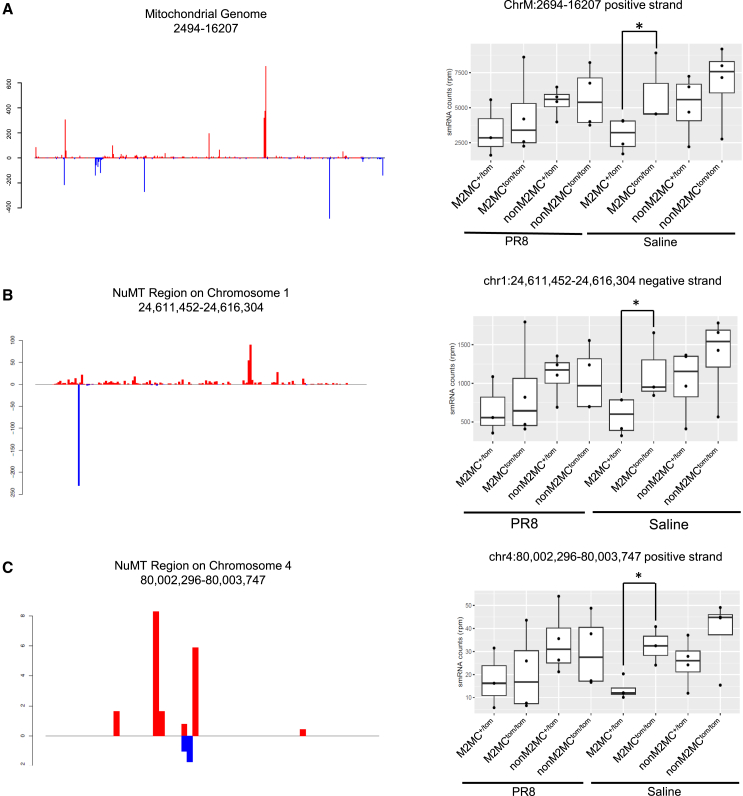
NUMT-derived small RNAs decrease in *Miwi2* expressing cells during homeostasis Representative coverage plots of small RNAs that aligned to the positive (red) and negative (blue) strands of the (A) mitochondrial genome and NUMT regions in (B) chromosome 1 and (C) chromosome 4. The most represented strands (positive/negative) were measured (right). Data are shown as a boxplot with the median (center line), interquartile range (IQR, box), and whiskers extending to min/max (or 1.5× IQR) (*Student’s t test*). ∗*p < 0.05*.

When examining host-derived small RNAs, a significant proportion were initially found to align with the mitochondrial genome in all multiciliated cell subtypes (Figures 5A and S8A). However, after increasing the alignment stringency, which ensures more precise matching, we could also match small RNAs from NuMT DNA in addition to the mitochondrial genome. NuMTs are genetic insertions of mitochondrial DNA in the nuclear genome and while their functional relevance remains debated, their expression has been linked to specific diseases such as cancer.^70^^,^^71^ Despite their poorly characterized role, these small RNAs mapped to specific loci of established NuMT regions on mouse chromosome 1 and 4 (Figures 5B, 5C, S8B, and S8C). The abundance of distinct NuMT-derived small RNA sequences, rather than broad expression across the entire NuMT region, suggests an active regulatory mechanism rather than random transcription or degradation. Notably, M2MC^+/tom^ cells exhibited significantly lower counts of these small RNAs compared to M2MC^tom/tom^ cells under saline conditions. Taken together, these findings suggest that *Miwi2* is associated with the downregulation of NuMT-derived small RNA expression in multiciliated cells.

### Enhanced mitochondrial reactive oxygen species in multiciliated cells of *Miwi2* deficient mice

**Figure 6:**
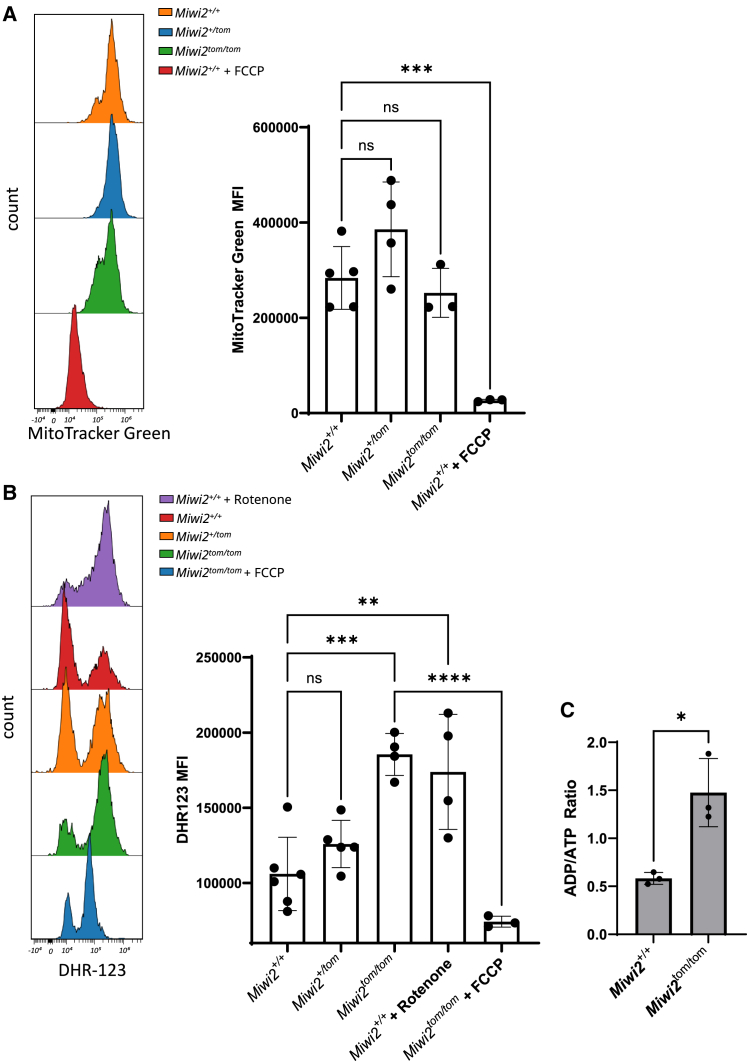
*Miwi2* deficiency increases intracellular ROS and ADP/ATP ratios in multiciliated cells but has no effect on mitochondrial mass during PR8 infection (A) Flow cytometry analysis assessing mean fluorescence intensity (MFI) of mitochondrial mass dye (MitoTracker Green) in PR8 infected multiciliated cells from *Miwi2*^*+/+*^ (*n* = 5), *Miwi2*^*+/tom*^ (*n* = 4), and *Miwi2*^*tom/tom*^ (*n* = 3) mice. As a negative control, *Miwi2*^*+/+*^ cells were treated with oxidative phosphorylation uncouple, FCCP, that dissipates the mitochondrial membrane. (B) MFI of ROS dye (DHR-123) was also assessed in infected multiciliated cells from *Miwi2*^*+/+*^ (*n* = 6), *Miwi2*^*+/tom*^ (*n* = 5), *Miwi2*^*tom/tom*^ (*n* = 4) mice. *Miwi2*^*+/+*^ cells (*n* = 4) were treated with mitochondrial complex I inhibitor, rotenone, and *Miwi2*^*tom/tom*^ cells (*n* = 3) were treated with FCCP as a positive and negative ROS control, respectively (*one-way ANOVA followed by Sidak’s test*). (C) ADP/ATP ratios of FACS isolated PR8 infected multiciliated cells from *Miwi2*^*+/+*^ (*n* = 3) and *Miwi2*^*tom/tom*^ (*n* = 3) mice (*Student’s t test*). Error bars represent mean ± standard deviation. ∗*p* < 0.05, *∗∗p < 0.005, ∗∗∗p < 0.0005, ∗∗∗∗p < 0.0001*.

Mitochondria undergo significant changes during viral infection such as elongation and increased oxidant generation.^72^^,^^73^ Given the previous observation that *Miwi2* could regulate mitochondrial genes during PR8 infection, we examined changes in mitochondrial features such as mass and intracellular ROS. PR8 infected *Miwi2*^*+/+*^, *Miwi2*^*+/tom*^, and *Miwi2*^*tom/tom*^ lungs were analyzed via flow cytometry. Using multiciliated cell surface markers, mitochondrial mass was assessed using mean fluorescence intensity (MFI) of MitoTracker Green staining.^74^ No *Miwi2* dependent changes in mitochondrial mass were observed during PR8 infection in multiciliated cells (Figure 6A).

Cells were separately stained with DHR-123, which targets mitochondrial ROS.^75^^,^^76^ Notably, DHR-123 MFI was significantly higher in multiciliated cells of *Miwi2*^tom/tom^ mice compared to *Miwi2*^*+/+*^ mice (Figure 6B). As a positive control for ROS generation, multiciliated cells from *Miwi2*^*+/+*^ mice were treated with mitochondrial complex I inhibitor rotenone that increased intracellular ROS.^77^ Conversely, in multiciliated cells from *Miwi2*^*tom/tom*^ mice, treatment with oxidative phosphorylation uncoupler FCCP significantly decreased intracellular ROS.^78^ Considering the association of mitochondrial oxidant generation with ATP production, we assessed ADP/ATP ratios in isolated PR8 infected multiciliated cells. Multiciliated cells of *Miwi2*^*tom/tom*^ mice exhibited a significant increase in ADP/ATP ratios compared to multiciliated cells of *Miwi2*^*+/+*^ mice (Figure 6C). These findings suggest that while *Miwi2* does not affect mitochondrial mass, it may alter mitochondrial energy dynamics and oxidant generation that can drive intracellular ROS during PR8 infection. Given the critical role of ROS production in the innate immune response, this alteration may influence the host’s ability to combat viral infections.

### *Miwi2* as a host susceptibility factor for influenza burden and pathogenesis

**Figure 7:**
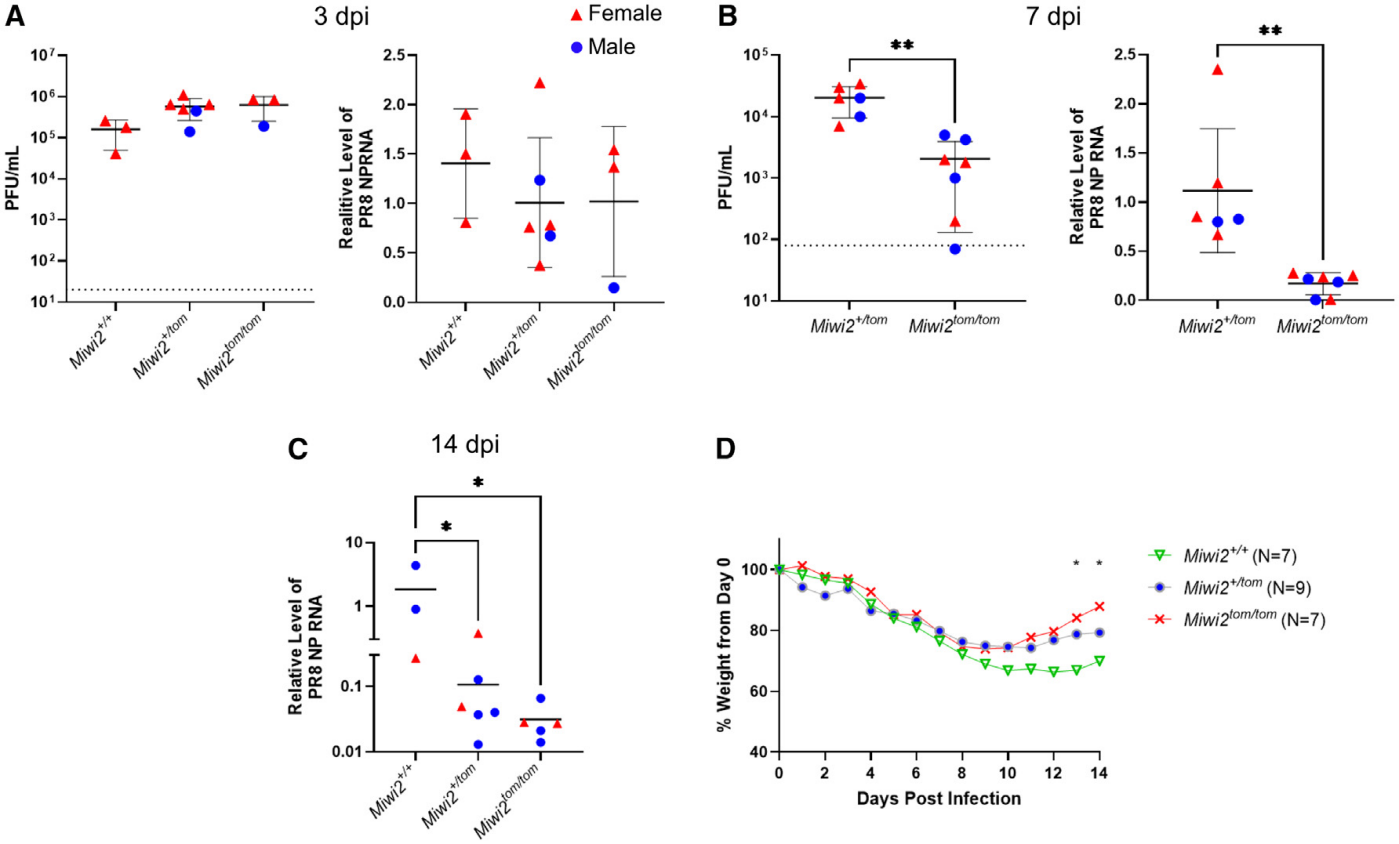
*Miwi2*^*tom/tom*^ mice experience decreased viral burden and weight loss (A) Quantification of plaque titers and relative expression of PR8 nucleoprotein (NP) RNA in *Miwi2*^*+/+*^ (*n* = 3), *Miwi2*^*+/tom*^ (*n* = 6), *Miwi2*^*tom/tom*^ (*n* = 3) lungs at 3 dpi. (B) The same analysis was performed on *Miwi2*^*+/tom*^ (*n* = 6), *Miwi2*^*tom/tom*^ (*n* = 7) lungs at 7 dpi (*Mann-Whitney U test)*. Dotted line represents initial administered PFU dose. (C) NP RNA was still detected 14 dpi in *Miwi2*^*+/+*^ (*n* = 3), *Miwi2*^*+/tom*^ (*n* = 6), *Miwi2*^*tom/tom*^ (*n* = 5) lungs (*one-way ANOVA followed by Dunnett’s test*). (D) Weight curves of PR8 infected mice measured every day until 14 dpi (*mixed-effects analysis*). Error bars represent mean ± standard deviation *∗p < 0.05, ∗∗p < 0.005.*

We next examined if *Miwi2* influences overall outcomes of influenza infection. *Miwi2*^*+/+*^, *Miwi2*^*+/tom*^, and *Miwi2*^*tom/tom*^ mice were infected at 3, 7, and 14 dpi. No significant changes in viral titers or RNA were observed at 3 dpi (Figure 7A). However, at 7 dpi, *Miwi2*^*tom/tom*^ lungs contained lower viral titers and RNA compared to *Miwi2*^*+/tom*^ lungs (Figure 7B). While viral titers are undetected at 14 dpi, viral RNA was still significantly lower in *Miwi2*^*tom/tom*^ and *Miwi2*^*+/tom*^ lungs compared to *Miwi2*^+/+^ (Figure 7C). When assessing weight, *Miwi2*^*tom/tom*^ mice recovered faster in bodyweight compared to *Miwi2*^*+/+*^ mice (Figure 7D). Although *Miwi2* deficiency mainly causes infertility in male mice, the observed differences in viral load were independent of sex.

It is possible that these differences in viral load could be attributed to differences in inflammation and immune cell recruitment in response to infection. To address this, we employed an immune cell flow cytometry panel for assessing immune cell recruitment in *Miwi2*^*+/+*^, *Miwi2*^*+/tom*^, and *Miwi2*^*tom/tom*^ mice at 3 dpi. No differences in CD8 T cell subsets or other immune cell types in recruitment were observed (Figure S9). Histopathological evaluations of all lung lobes from PR8 infected lungs revealed no significant morphological differences between *Miwi2*^*tom/tom*^ and *Miwi2*^*+/+*^ mice either at 3 dpi or at 7 dpi (Figure S10). While *Miwi2*^*tom/tom*^ mice experience improved outcomes to IAV infection, these findings suggest that it is not due to alterations in immune cell recruitment and histopathological patterns.

## Discussion

MIWI2, the mouse ortholog of human PIWIL4, is an argonaute family protein highly expressed in the mammalian germline, where it plays a critical role in maintaining genomic integrity. Although somatic expression of *Miwi2* has been observed, its role in mammalian somatic cells has remained largely unexplored.^79^^,^^80^^,^^81^ In this study, we uncover a previously unrecognized role for *Miwi2* in the host defense against IAV infection, specifically in multiciliated cells of the respiratory tract. Unlike the canonical role of Piwi proteins regulating retrotransposons in the male germline and viral RNA in insects, *Miwi2* appears to modulate select mitochondrial genes during viral infection through regulation of NuMT-derived small RNAs. Additionally, *Miwi2* deficient multiciliated cells exhibited increased intracellular ROS levels and ADP/ATP ratios during IAV infection, and *Miwi2* deficient mice show reduced morbidity and viral load. These findings suggest that *Miwi2* plays a more dynamic and context-dependent role in somatic cells, particularly during stress conditions such as viral infections, where it may influence mitochondrial function and host-pathogen interactions.

Multiciliated cells are highly metabolically active, largely due to the energy demands of ciliary beating.^82^ These cells often undergo mitochondrial adaptations in response to stress, such as viral infections, which include mitochondrial elongation and changes in ROS production.^72^^,^^73^^,^^83^ Our study demonstrated increased intracellular ROS in multiciliated cells from *Miwi2* deficient mice during early IAV infection, while mitochondrial mass remained unchanged. The precise mechanism through which *Miwi2* or the M2MC population influences mitochondrial energy dynamics and increased ROS in all multiciliated cells remains unclear. However, *Miwi2* deficiency appears to contribute to more efficient viral clearance, as evidenced by reduced viral titers and RNA at later stages of infection.

We observed an increase in ADP/ATP ratios in multiciliated cells of *Miwi2* deficient mice. This could be a result of slower ADP-to-ATP conversion or rapid ATP consumption. If the former, *Miwi2* deficiency may impair mitochondrial function, as genes encoding complexes I, III, and V were downregulated during IAV infection (Table S1). Inhibiting these complexes, such as with rotenone, disrupts electron transport, increasing ROS production while reducing ATP.^84^^,^^85^^,^^86^ Rotenone treatment has been shown to normalize oxygenation and airway resistance and reduce IAV-induced pulmonary edema.^87^ Alternatively, if ATP consumption is driving the increased ADP/ATP ratios, *Miwi2* may promote mitochondrial hyperactivity during infection, further elevating ROS.

ROS generation, which is closely associated with mitochondrial function and reactive nitrogen species (RNS) generation, plays a complex role in multiciliated cells. While excess oxidant generation can lead to oxidative stress and tissue damage, moderate RNS levels, such as nitric oxide, are essential for maintaining planar cell polarity and ciliary beat frequency, both of which are necessary for effective airway clearance.^82^^,^^88^^,^^89^^,^^90^^,^^91^ Influenza infection impairs ciliary beating and damages cilia, compromising mucociliary clearance and thus facilitating viral entry and propagation.^92^^,^^93^ Therefore, it is possible that *Miwi2* deficiency enhances the resilience or effectiveness of ciliary function during infection, potentially contributing to the improved antiviral host defense.

Increased intracellular ROS can promote cellular apoptosis, a mitochondrial-mediated cell death pathway crucial for regulating viral infections. Previous studies have demonstrated that endoplasmic reticulum stress induces piRNA production and that PIWIL4 expression drives apoptosis in human airway epithelial cells.^94^ In our study, we observed a decrease in M2MC cells in *Miwi2* heterozygous mice compared to deficient mice during PR8 infection (Figure 1E), suggesting that *Miwi2* may trigger apoptosis in infected cells potentially contributing to further tissue injury. Moreover, mitochondrial ROS can activate the innate immune response to infection.^95^^,^^96^ Although we did not observe differences in immune cell recruitment during the early stages of infection, alterations in ROS levels could influence the functional efficiency of immune cells in clearing the virus. Future studies are needed to determine the specific mechanisms by which *Miwi2* influences viral clearance.

The evolutionary significance of maintaining a subset of multiciliated cells expressing *Miwi2*, may stem from its potential role in regulating mitochondrial function, mitigating excessive oxidative damage, and regulating inflammatory responses. While *Miwi2* deficiency results in increased ROS levels, which could enhance antiviral defenses, sustained ROS elevation poses risks of prolonged oxidate damage. This could exacerbate inflammatory responses and compromise tissue repair, especially in chronic infection or inflammatory conditions.^97^ By tempering ROS levels in some contexts, *Miwi2* may serve to balance between effective pathogen clearance and the prevention of excessive oxidative damage, highlighting its potential role in preserving long-term lung homeostasis and function.

MIWI2 has been extensively studied for its role in silencing retrotransposons in the mammalian male germline. Previous RNA-sequencing studies have suggested viral infections may re-activate transposable elements, and promote immune defenses by regulating expression of nearby antiviral genes or can act as immunostimulatory self-derived viral mimetics to trigger defenses.^64^^,^^98^ Our findings indicated that IAV infection induces expression of several retrotransposons subfamilies, specifically ERVs, in airway multiciliated cells. ERVs have genetic structures similar to exogenous retroviruses and, upon activation, generate double-stranded RNA species than can be detected by cytosolic RNA sensors such as RIG-I, MDA5, or toll-like receptors.^99^ The reactivation of these ERV subfamilies could be a method of strengthening the antiviral response to IAV infection. Despite these findings, their expression does not appear to be directly associated with *Miwi2* expression. However, more RT subfamilies are expressed in multiciliated cells of *Miwi2* deficient mice, including nonM2MC cells. Therefore, it is plausible that *Miwi2* alters the cellular state of multiciliated cells that may facilitate increased RT activation during influenza infection through an unknown mechanism.

Viral-derived small RNAs, such as miRNAs from IAV, have been documented to regulate viral RNA synthesis and act as molecular switches from viral transcription to replication.^100^ Similarly, in SARS-CoV models, small RNAs derived from the nsp3 and N genomic regions contribute to viral pathogenesis.^101^ Our data are thus consistent with previous studies where we can detect significant amounts of viral-derived small RNAs in influenza targeted multiciliated cells. Although the production of these small RNAs is independent of *Miwi2* expression, their role in viral replication and pathogenesis remains to be determined and may represent another layer of host-pathogen interaction in infected multiciliated cells of the airways.

NuMT-derived small RNAs were also identified in multiciliated cells. NuMTs are fragments of mitochondrial DNA that have integrated into the nuclear genome throughout evolutionary history.^70^^,^^102^ Some studies suggest they are ancient evolutionary insertion events with no observed function, while others have associated them with genomic instability and cancer.^103^^,^^104^^,^^105^ Although the role of NuMTs in gene regulation is unclear, the selective regulation of NuMT RNAs rather than random degradation, hints at an organized, potentially piRNA-like mechanism in somatic cells that targets either these mitochondrial DNA insertions or their transcripts for post-transcriptional repression.

Our observations revealed a decrease in the NuMT-derived small RNAs in *Miwi2* expressing cells at baseline, indicating that *Miwi2* may regulate their biogenesis or turnover during homeostasis. This gene-dependent regulation of NuMT-derived small RNAs has not been reported before, highlighting a novel aspect of *Miwi2*’s role in somatic cells. Interestingly, although we did not observe *Miwi2*-dependent changes in mitochondrial mRNAs in saline treated M2MC cells, it is possible that the functional significance of these small RNAs only becomes evident during cellular stress such as during viral infection, when the regulation of mitochondrial function is more critical.^106^ One particular observation is that primary piRNA biogenesis occurs near the mitochondria, which are localized proximal to the basal body of multiciliated cells. This is coincidentally where MIWI2 is also localized.^23^^,^^33^^,^^35^ Given the repressive nature of piRNAs, it is conceivable that NuMT transcripts are directed to the mitochondria, where they are processed by MIWI2 to generate piRNAs. This spatial proximity may facilitate the regulation of mitochondrial-nuclear communication, potentially influencing both mitochondrial function and the broader stress response pathways in multiciliated cells. Moreover, if these small RNAs are indeed involved in stress responses, particularly in regulating mitochondrial activity, they could play a critical role in the host antiviral defense mechanisms. Further investigation is needed to elucidate the functional impact of the NuMT-derived small RNAs and to interrogate their novel gene regulatory properties influencing both mitochondrial function and host defense pathways.

In conclusion, our findings suggest that *Miwi2/Piwil4* acts as a potential host susceptibility factor for severe respiratory infections. These studies highlight a novel function of somatic *Miwi2* in the lung, relevant during IAV infection. By regulating mitochondrial gene expression and function, *Miwi2* may exacerbate influenza disease through its impact on intracellular ROS levels and ATP production in multiciliated cells. Further elucidation of this unique somatic *Miwi2*-dependent pathway could reveal potential therapeutic targets designed to mitigate the severity of viral respiratory infections and enhancing host defense.

### Limitations of the study

A limitation of this study is its focus on an early stage of infection, which may not capture the full dynamics of *Miwi2*’s role over the complete course of infection. Moreover, the specific molecular pathways linking *Miwi2* to mitochondrial function, ROS production, and immune response modulation remain to be elucidated. Finally, our *Miwi2*-deficient model was a systemic knockout, so while the changes in influenza disease burden are likely attributable to the M2MC cells, the possibility that *Miwi2* expression in other cells and tissues contributes to IAV pathogenesis cannot be excluded. Despite these limitations, our findings yield novel insights into the role of *Miwi2* and small RNAs in multiciliated cells during influenza infection.

## Resource availability

### Lead contact

Further information and requests for resources should be directed to and will be fulfilled by the lead contact, Jhonatan Henao Vasquez (jhenao@bu.edu).

### Materials availability

This study did not generate new materials.

### Data and code availability

Bulk small and long RNA sequencing files have been deposited in the NCBI Gene Expression Omnibus (GEO) under accession number GEO: GSE276578. Any additional information required to reanalyze the data reported in this paper is available from the lead contact upon request. No original code or software was used in this study.

## Acknowledgments

This work was supported by 10.13039/100000002NIH F31-HL165892 (to J.H.V.), 10.13039/100000002NIH T32-HL7035 (to J.H.V.), 10.13039/100000002NIH F32-HL160094 (to J.Y.), 10.13039/100000002NIH R01-HL136725 (to M.R.J. and A.F.), AHA 23PoST1022559 (to E.C.), FY24 Department of Medicine Research Accelerator Program (to E.C.), 10.13039/100000002NIH R01-GM135215 (to N.C.L.), 10.13039/100000002NIH R01-AR078306 (to N.C.L.), and 10.13039/100000002NIH R01-AG078930 (to N.C.L.). The authors like to thank the members of the Boston University Flow Cytometry Core (Anna C. Belkina and Brian Tilton) and Tufts University Genomics Core (Albert K. Tai and Irina V. Grinvald) for technical support on FACS and RNA sequencing, respectively. The Pneumonia Biology Labs (Joseph P. Mizgerd, Katrina E. Traber, Lee J. Quinton, and Paul J. Maglione) of the Pulmonary Center at Boston University provided invaluable feedback on this work. Figures 1A and 1C (https://BioRender.com/p22a865) and graphical abstract (https://BioRender.com/f53y021) were generated using BioRender.

## Author contributions

J.H.V., J.Y., A.F., and M.R.J. conceived and designed the experiments. J.Y. performed influenza RT-qPCR, plaque assay, and weight loss studies. C.J.L. provided small RNA analysis assistance. N.C.L. provided funding, analysis, and guidance to C.J.L.. V.A.D. and F.S. provided western blot analysis of sorted epithelial cells. M.L. provided histopathological evaluation of influenza infected lungs. J.H.V. performed and analyzed all other experiments. J.L.F. provided guidance on developing the protocol for the flow cytometry evaluation of mitochondrial mass and ROS. J.P.M., A.F., and M.R.J. contributed to reagents and materials. J.H.V., A.F., and M.R.J. wrote the manuscript with contributions from all authors.

## Declaration of interests

The authors declare no competing interests.

## STAR★Methods

### Key resources table

**Table:** 

REAGENT or RESOURCE	SOURCE	IDENTIFIER
**Antibodies**		

α-CD326 APC (Clone G8.8)	BioLegend	118214; RRID: AB_1134102
α-CD45 PE-Cy 7 (Clone 30-F11)	BD Biosciences	552848; RRID: AB_394489
α-CD24 Pacific Blue (Clone M1/69)	BioLegend	101820; RRID: AB_572010
7-AAD	BD Biosciences	559925
α-CD16/CD32 (Fc block)	BioLegend	101302; RRID: AB_312800
α-Ly6G FITC (Clone 1A8)	Abcam	Ab25024 RRID: AB_470400
α-CD45 BV510 (Clone 30-F11)	BioLegend	103138 RRID: AB_2561392
α-Ly6C eFlour 450 (Clone HK1.4)	Invitrogen	48593280; RRID: AB_10805518
α-NKp46 PerCP-Cy5 (Clone 9E2)	BioLegend	331919; RRID: AB_2561665
α-CD4 APC-Cy7 (Clone GK1.5)	BD Biosciences	552051; RRID: AB_394331
α-CD8 Dazzle 594 (Clone SK1)	BioLegend	344743; RRID: AB_2566515
α-CD69 eFluor 450 (Clone H1.2F3)	eBioscience	48-0691-82; RRID: AB_10719430
α-CD3 PerCP-Cy5.5 (Clone 17A2)	Biolegend	100217; RRID: AB_1595492
α-CD103 PE-Cy7 (Clone 2E7)	BioLegend	121425; RRID: AB_2563690
α-CD44 APC (Clone IM7)	BioLegend	103011; RRID: AB_312962
α-CD62L AC-Cy7 (Clone MEL-14)	BioLegend	104427; RRID: AB_830798
α-CD45.2 FITC (Clone 104)	Biolegend	109805; RRID: AB_313442
α-nucleoprotein	ThermoFisher	PA5-32242; RRID: AB_2549715
α-LINE-1 ORF1p	Abcam	Ab216324; RRID: AB_2921327
α-GAPDH	Sigma	G9545; RRID: AB_796208

**Bacterial and virus strains**		

Mouse Adapted A/Puerto Rico8/8-1-MA/1934(H1N1)	Harding, A. T. et al.^50^	N/A

**Chemicals, peptides, and recombinant proteins**		

MitoTracker Green	ThermoFisher	M46750
Dihydrorhodamine 123	ThermoFisher	D23806
Rotenone	MilliporeSigma	5573681 GM
FCCP	Tocris	Bioscience

**Critical commercial assays**		

ADP/ATP Bioluminescence Kit	Sigma-Aldrich	MAK135
TaqMan™ RNA-to-C_T_™ 1-step kit	Applied Biosystems	4392938
RNeasy Mini Kit	Qiagen	74104
QIAsymphony RNA Kit	Qiagen	931636

**Experimental models: Cell lines**		

Canine: MDCK Cells	ATCC	CCL-34

**Experimental models: Organisms/strains**		

C57BL/6 *Miwi2*^*+/tom*^, *Miwi2*^*tom/tom*^ mice	Carrieri et al.^48^	N/A

**Oligonucleotides**		

PrimeTime qPCR probe (IAV NP) 5'-/56-FAM/AGG CACCAA/ZEN/ACG GTC TTA CGA ACA/31ABkFQ/-3'	Integrated DNA Technologies	280479528
PrimeTime qPCR Forward Primer (IAV NP) 5′-CGT TCT CCA TCA GTC TCC ATC-3′	Integrated DNA Technologies	280474883
PrimeTime qPCR Reverse Primer (IAV NP) 5′-GAG TGA CAT CAA AAT CAT GGC G-3′	Integrated DNA Technologies	280474884

### Experimental model and study participant details

#### Study approval

All mouse experiments were performed in accordance with US federal law and approved by the Boston University Chobanian and Avedisian School of Medicine IACUC (Permit 14859).

#### Mice

*Miwi2*^*+/tom*^ and *Miwi2*^*tom/tom*^ mice were provided by Dr. Donal O’Carroll from the University of Edinburgh and housed in the Boston University Animal Science Center on a 12-hour light-dark cycle with access to food and water *ad libitum*. *Miwi2*^*+/+*^ were generated from dual *Miwi2*^*+/tom*^ breeding pairs. The sex of the mice was randomized across experimental groups and were 8-12 weeks of age. Mice were ethically euthanized using isoflurane followed by inferior vena cava incision at time of sacrifice. All animal experiments were performed in compliance with approved Boston University animal protocols.

#### Influenza stock

Mouse-adapted A/Puerto Rico8/8-1-MA/1934(H1N1) (PR8) was propagated as described previously.^50^^,^^107^ Specific pathogen free fertilized chicken eggs (Charles River) were incubated at 37.5°C at 60% relative humidity for 10 days while rotating every 180 minutes. Eggs were candled every 5 days to inspect for healthy embryo development. At day 10, a centerpunch was used to puncture a hole in the shell into the air sac right above the allantoic fluid. 100 PFU of PR8 stock was inoculated into the allantoic fluid via the air sac and hole was sealed with hot glue. Eggs were incubated for an additional 3 days (no rotation) at 35°C and then place in 4°C overnight. Allantoic fluid was then harvested and pooled. Viral titer was determined via plaque assay. Lethal dose 50 of intratracheal and intranasal instillations of every new PR8 batch was performed on sex matched 8-week C57BL/6 mice to determine appropriate sublethal dose for experiments.

### Method details

#### Experimental infections

PR8 lung infections were performed via intratracheal and intranasal route. Mice were anesthetized by intraperitoneal injection of ketamine (50 mg/kg) and xylazine (5 mg/kg) diluted in sterile saline. For intratracheal instillations, a 24-gauge angiocatheter was inserted into the trachea and directed into the left bronchus. 50μL bolus of 20-100 PFU of PR8 in sterile saline was instilled followed by a bolus of air. Mice were placed in the right lateral decubitus position. For intranasal instillations, mice were held upright while 25μL of 200 PFU was given dropwise into the left nostril. Again, mice were place in the right lateral decubitus position.

#### Lung digest and epithelial enrichment for FACS

Lungs were digested as described previously.^108^ Lungs were perfused with 10 mL of HBSS and later washed with 10 mL of 5 mM EDTA in DPBS, 1 mL of 10 mg/mL DNAse in PBS, and then inflated with 1mL of elastase and DNase cocktail in 10% dextran in RPMI 1640 with 0.5mL 1% low melt agarose. Heart and lungs were placed on a small petri dish containing 2mL of the elastase/DNase cocktail and place on a rotator at 100 rpm for 45 minutes. Afterwards, the left lung lobe was minced, transferred to a conical, and incubated on the shaker at 37°C at 100rpm for 5 minutes. 20mL of 50:50 fetal bovine serum:RPMI 1640 and additional DNase was added to the conical and incubated for 5 minutes at 37°C at 300xg. Samples were sequentially filtered in 100μm, 70μm, and 40μm sized cell-strainers and centrifuged for 10 minutes at 4°C at 300 x g. Supernatant was removed and resuspended in 1 mL of red blood cell lysis buffer for exactly 1 minute and then quenched in 9mL of PBS. Cells were centrifuged for 5 minutes at 4°C at 300 x g and resuspended in FACS buffer (0.5% FBS, 2mM EDTA in PBS) with α-CD16/CD32 in a final concentration of 10^7^ cells/mL. Cells were stained with 1:100 APC α-CD326 (BioLegend 118214), 1:100 PE-Cy 7 α-CD45 (BD Biosciences 552848), and 1:100 Pacific Blue α-CD24 (BioLegend 101820) and 7-AAD (BD Biosciences 559925).^23^ Cells were sorted on a Beckman Coulter MoFlo Astrios. Flow plots were generated and analyzed using OMIQ and fluorescence minus one were generated to confirm positive population (Figure S11A). M2MC cell frequency ranges from 1000-5000 cells per left lobe (Figure S11B). In order to ensure enough RNA content is collected for sequencing, 4-6 left lobes of genotype and treatment matched mice were pooled prior to sort.

#### Library preparation and small and long RNA sequencing

TdTomato+ and tdTomato- multiciliated cells were sorted into 500μL of Qiagen buffer RLT supplemented with 1% β-mercaptoethanol and then flash frozen in liquid nitrogen. Samples were stored in -80°C until remaining samples were collected. Total RNA was isolated on the Qiagen QIASymphony SP/AS using the QIAsymphony RNA kit and the miRNA CT 400 protocol. RNA content and integrity was measured using the Agilent Bioanalyzer. RNA Quality Number (RQN) of > 8 was considered acceptable RNA quality for sequencing. Samples that were below the RQN were discarded, and replacement samples were collected. The Qiagen miRNA and Illumina Stranded Total RNA with Ribo Zero Plus kits were used to construct the small and long RNA libraries respectively per manufacturer’s protocol. Small RNAs were sequenced on the NextSeq 550 at high-output single-end 75 cycles. Long RNAs were sequenced on the NovaSeq S1 flow cell at single-end 100 cycles.

#### Bioinformatics of coding gene expression

Base calling, demultiplexing and fastq file generation was performed with Illumina bcl2fastq. Reads were mapped to mouse reference genome mm10, mouse-adapted Influenza A virus (A/Puerto Rico8/8-1-MA/1934(H1N1)), and antibiotic resistance plasmid containing tdTomato (Table S2) using HISAT2, and count table generated with feastureCounts of the SubRead package. The count table is then used as input for differential expression analysis using DESeq2. Differentially expressed genes were analyzed using Enrichr, with pathway enrichment performed against the BioPlanet 2019 database.^109^ This approach facilitated the identification of relevant biological pathways associated with the observed gene expression changes.

#### Bioinformatics of piRNA, viral RNA, and RT expression analysis

Small and total RNA sequencing reads were downloaded from Tufts Medical Center FTP server. Reads were mapped to mouse GRCm38/mm10 (GCA_000001635.2) and Influenza A Puerto Rico 8 virus (GCA_000865725.1) genomes using bioinformatic pipeline MSRG.^51^^,^^52^^,^^110^ Small RNA-seq reads quality was checked using length distribution plots (peak at 22 nucleotides) generated from MSRG. Samples PR8 M2MC^+/tom^ replicate 1 and Saline M2MC^tom/tom^ replicate 2 from small RNA-seq were discarded from downstream analysis due to low quality. Read counts mapped to PR8 virus were recorded and visualized in coverage plots. One-tailed T-tests were conducted between heterozygous vs deficient, M2MC vs nonM2MC, and PR8 vs Saline in PR8 virus expression.

For analysis of RT and viral derived long RNAs, total RNA-seq reads quality were checked using FastQC. Total RNA-seq reads were trimmed to 35 nucleotides and run though MSRG pipeline with the addition of NUMT sequences (showed expressions from the previous analysis) loaded. One-tailed T-tests were conducted between the genotypes in NUMTs expressions. Reads mapped to the two abundant transposons in mice L1MdTf_I, RLTR4I_MM_ERV1, and IAPLTR1a_MM_ERV2 were compared between heterozygous vs deficient. RPM counts per mouse TE were determined from the input of each replicate of RNA-seq library reads into the MSRG pipeline.^52^ TE entries were then sorted and kept for analysis only if the base mean expression was greater than 2 RPMs (Log2>1).

#### Bioinformatics for miRNAs

For miRNAs, raw sequencing data is first processed using a custom pipeline and script to remove adaptor sequence, remove invalid and consolidate UMI, and remove reads that are shorter than 15 bases. The processed reads are then use as input for COMPSRA analysis. Differential expression analysis were than performed on the output using R and DESeq2 package comparing M2MC vs nonM2MC samples.

#### Lung digestion for leukocyte flow cytometry

Lungs were collected with RPMI 1640 with 10% FBS before processing for flow cytometry as described previously.^111^ Single cell suspensions were prepared by digestion of lungs in type 2 collagenase (Worthington Biochemicals, Lakewood, NJ) and DNAase I. Cells were blocked with αCD16/CD32 Fc-Block (Biolegend). Flow cytometry was performed on LSR II Flow cytometer (BD Biosciences). Cells were stained with 7-AAD, FITC α-Ly6G (abcam), BV510 α-CD45 (BioLegend), eFlour 450 α-Ly6C (eBioscience), PerCP-Cy5 α-NKp46 (BioLegend), APC α-CD64 (BioLegend), APC-Cy7 α-CD4 (BD Biosciences), PE-Dazzle 594 α-CD8 (BioLegend). Established surface markers were used to categorize the following CD45 subsets^112^^,^^113^^,^^114^^,^^115^^,^^116^ (Figure S12).

#### CD8 T cell panel for flow cytometry

Mice were anesthetized via intraperitoneal injection, as stated previously, and injected retro-orbitally with 2 μg of IV FITC α-CD45.2 (BioLegend) antibody. This was allowed to circulate for 3 minutes prior to sacrifice. Mediastinal lymph nodes (LNs) were collected from influenza infected mice and placed in 1 mL of cold PBS. LN tissue was passed through a 100μm strainer which was then washed with 3 mL of cold FACS buffer. Lungs were processed for leukocyte flow cytometry as stated above. Cells were stained with eFluor 450 CD69 (eBioscience), PerCP-Cy5.5 CD3 (Biolegend), PE-Cy7 CD103 (BioLegend), APC CD44 (BioLegend), and APC-Cy7 CD62L (BioLegend) for 30 minutes on ice. Excess antibody was washed with 3mL of FACS buffer and centrifugation at 300xg at 4°C for 5 minutes. Cell pellets were resuspended in 300 μL FACS buffer with 5 μL 7-AAD (BD Biosciences). Cells were gated using established markers^112^^,^^117^^,^^118^^,^^119^ (Figure S13).

#### Flow cytometry analysis of mitochondrial mass and intracellular ROS

Single-cell suspensions were prepared as mentioned above. Each sample was split into two separate subsamples each containing 10^6^ cells. For staining controls, cells suspensions were treated with 50μM FCCP and 0.5 μM rotenone for 10 minutes prior to staining incubation. All samples were stained for the same multiciliated cell markers as described above. The subsamples were additionally incubated with either 100nM MitoTracker or 2.5μM DHR-123 to examine mitochondrial mass and ROS, respectively. Staining concentrations were determined by previous titer experiments (Figure S14). Stained samples containing DHR-123 were incubated at 37°C for 30 minutes and stained samples containing MitoTracker were incubated on ice for 30 minutes. After incubation, cells were washed with PBS, centrifuged at 300xg, and resuspended in FACS buffer supplemented with 7-AAD and analyzed on the Cytek Aurora.

#### qRT-PCR

RNA isolation from mouse whole lung homogenate was performed using the Qiagen RNeasy mini kit according to the manufacturer’s instructions. qRT-PCR was performed using the TaqMan and QuantStudio 3 (Applied Biosystems 4392938) according to the manufacturer’s instructions. PR8 nucleoprotein qRT-PCR was performed using forward primer 5′-CGTTCTCCATCAGTCTCCATC-3′, reverse primer 5′-GAGTGACATCAA AATCATGGCG-3′, and probe 5'-/56-FAM/AGGCACCAA/ZEN/ACGGTCTTACGAACA/31ABkFQ/-3'.

#### Influenza plaque assay

Left lung lobes from PR8 infected mice were minced using a sterile razor blade in a petri dish, transferred to a bullet blender tube in 2 mL of viral grown media (as below) supplemented with 1 1μg/mL TPCK trypsin, and homogenized. Cell debris was pelleted by centrifugation at 5,000xg fat 4°C for 5 minutes, and the supernatant was collected and used to infect MDCK cells for 1 hour. After an hour, inoculum was removed and MDCK cells were incubated in 1 part 2X viral growth media, and 1 part 2.4% avicel supplemented with 1 μg/mL TPCK trypsin. Plaques were enumerated after 3 days.

#### Viral growth media (500mL)

a. 475mL of DMEM (ThermoFisher ref 11995-065).
b. 5mL Pen/Strep 100x (ThermoFisher 15140122).
c. 12.5mL of 1M HEPES (Sigma-Aldrich H0887).
d. 5mL Glutamax 100x (ThermoFisher 35050061).
e. 3mL of 35% BSA stock (Sigma-Aldrich A7409) OR 3.3ml of 30% BSA.

#### Western Blot

Whole lungs were lysed in protein extraction buffer (1M Tris pH 7.4, 5M NaCl, 0.5% sodium deoxycholate, 2% NP-40, 0.2% SDS) by bullet blender. Epithelial cells were sorted directly into protein extraction buffer. Protein sample was combined with 4X NuPage LDS buffer and reducing agent (Life Technologies). Samples were then heated for 10 minutes at 70°C before loading onto a 4%–12% Bis-Tris gel in mini cell with the inside buffer chamber filled with 1X running buffer and NuPage antioxidant (Life Technologies) and the outer buffer chamber with 1X running buffer with antioxidants. Protein was then transferred onto an Immobilon-P PVDF membrane (Millipore) using the X-Cell Blot II system. Membranes were separately probed with rabbit anti-nucleoprotein (Thermo, PA5-32242), rabbit anti-GAPDH (Sigma, G9545), and rabbit anti-ORF1p (Abcam, ab216324) followed by anti-rabbit IgG-HRP (Cell Signaling Technology) and developed with ECLPlus (GE Healthcare) before exposure to film (GE Healthcare). After each antibody stained, the membrane was stripped using Re-Blot plus for 20 minutes prior to staining with the next antibody.

#### ADP/ATP luciferase assay

Multiciliated cells of 3dpi PR8 *Miwi2*^*+/+*^ and *Miwi2*^*tom/tom*^ were isolated via FACS using the same markers described above and into 1 X PBS supplemented with 2.5% FBS and 2mM EDTA (FACS buffer). 10^3^ cells were aliquoted from each sample at a 10μL volume to assess ADP/ATP ratios using a bioluminescence kit (Sigma-Aldrich MAK135) per manufacturer’s instructions. Cells were incubated in ATP reagent for 1 minute in a clear-bottom 96-well plate prior to recording the first relative light units (RLU_A_) on the Synergy LX multi-mode reader set to the default luminescence protocol. After 10 minutes, the plate was recorded a second time to obtain the RLU_B_. ADP reagent was added to each sample and incubated for 1 minute prior to recording a third time (RLU_C_). ADP/ATP ratios were calculated using the equation below. Blank controls(10μL of FACS buffer with no cells) were used confirm RLU values were above detection.

$$\frac{ADP}{ATP}=\frac{{RLU}_{C}-{RLU}_{B}}{{RLU}_{A}}$$

#### Tissue processing, H&E staining and histopathology scoring

Lungs were harvested and fixed in 10% neutral buffer formalin for 24 hours and transferred to 70% ethanol. The fixed lung tissues were processed in a Tissue-Tek VIP-5 automated vacuum infiltration processor (Sakura Finetek USA, Torrance, CA, USA), followed by paraffin embedding with a HistoCore Arcadia paraffin embedding machine (Leica, Wetzlar, Germany) to generate formalin-fixed, paraffin embedded (FFPE) blocks, which were sectioned to 5 μm, transferred to positively charged slides, deparaffinized in xylene, and dehydrated in graded ethanol. Sections were stained with hematoxylin and eosin stain (H&E), dehydrated and cover slipped. Semi-quantitative histopathology analysis was conducted by a board-certified pathologist (ML) based on the following histopathology criteria:

(1) Presence (+1) or absence (+0) of mild to moderate necrotizing bronchiolitis, with remodeling of bronchiolar epithelium (attenuations with or without hyperplasia and regeneration of bronchiolar epithelial cells.
(2) Presence (+1) or absence (+0) of mild to moderate, neutrophilic and mononuclear infiltration of bronchiolar mucosa and in the peribronchiolar regions surrounding the bronchioles.
(3) Presence (+1) or absence (+0) of peribronchiolar and perivascular (medium to large-caliber vessels adjacent to bronchioles) mononuclear infiltrates.
(4) Absent (+0), minimal (+1), mild (+2), and moderate (+3) alveolar neutrophilic and mononuclear infiltrates.
(5) Absent (+0), minimal (+1), mild (+2), and moderate (+3) perivascular (small-caliber and medium caliber vessels in the pulmonary parenchyma) and adjacent interstitial (capillaries in the alveolar septa) neutrophilic and mononuclear infiltrates, with endothelial leukocyte adhesion and transmigration.

### Quantification and statistical analysis

Measured NuMT data are represented as boxplots and all other pooled data are presented as mean ± standard deviation with the number of biological replicates indicated in the figure legend. Statistical analyses were performed using GraphPad Prism and the type of analysis performed is denoted in the figure legend. “n” represents an individual mouse left lung, except for the RNA-sequencing studies were “n” represents 4-6 pooled mouse left lungs. Comparisons between 2 groups were performed using a 2-tailed unpaired student’s *t* test in the case of data shown to be parametric via the Shapiro-Wilk test. If the data was non-parametric, a Mann-Whitney U test was used. Comparisons between multiple groups were performed using 1-way or 2-way ANOVA with an appropriate post-hoc test denoted in the figure legend. To control for multiple comparisons in RNA-Seq data, FDR was calculated (*p*_adj_). Comparisons were considered significant if *p*_*adj*_<0.05. For mice weight trends, mixed-effects analysis was performed to control for multiple comparisons.
