# Supplementary material for: Somatic Miwi2 modulates mitochondrial function in airway multiciliated cells and exacerbates influenza pathogenesis
Source: iScience. 2025 Mar 25;28(4):112291. doi: 10.1016/j.isci.2025.112291 (PMC12002665; doi:10.1016/j.isci.2025.112291)
Supplement: Document S1. Figures S1–S14 and Tables S1 and S2 [file mmc1.pdf]

iScience, Volume *28*

Supplemental information

Somatic *Miwi2* modulates mitochondrial function in airway multiciliated cells and exacerbates influenza pathogenesis

Jhonatan Henao Vasquez, Jin Yuan, Chi Jing Leow, Erin Crossey, Fengzhi Shao, Senegal Carty, Viviana A. Dominguez, Ming Lo, Joseph P. Mizgerd, Jessica L. Fetterman, Nelson C. Lau, Alan Fine, and Matthew R. Jones

Figure S1

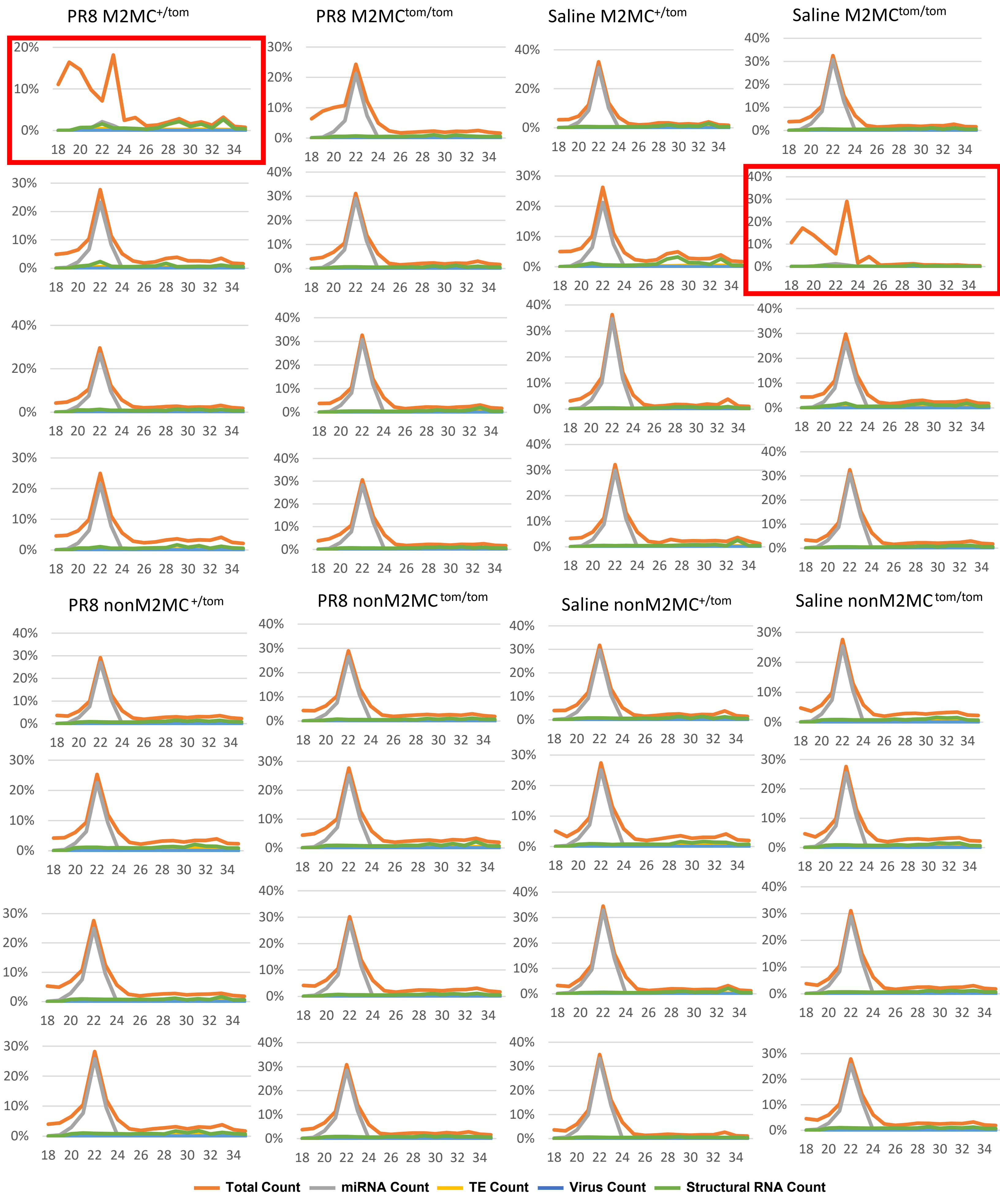
PR8 M2MC+/tom
PR8 M2MCtom/tom
Saline M2MC+/tom
Saline M2MCtom/tom
PR8 nonM2MC+/tom
PR8 nonM2MCtom/tom
Saline nonM2MC+/tom
Saline nonM2MCtom/tom
Total Count
miRNA Count
TE Count
Virus Count
Structural RNA Count

Figure S2

A

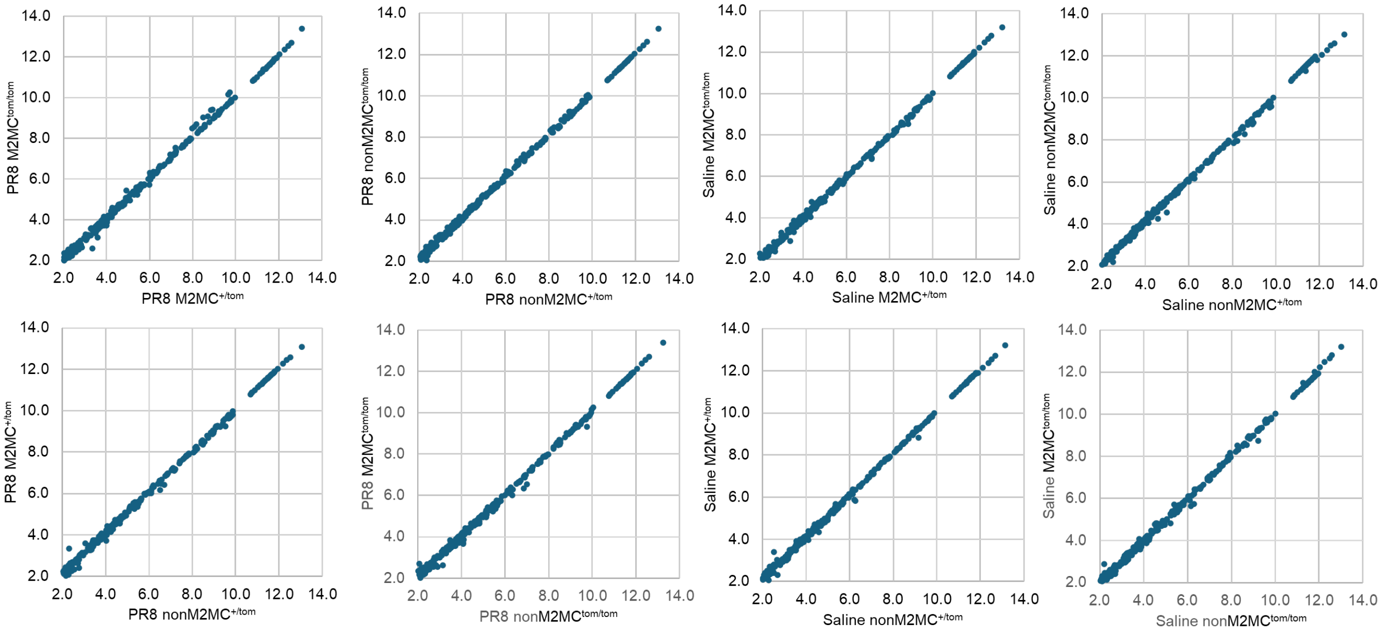
PR8 M2MC$^{tom/tom}$
PR8 M2MC$^{+/tom}$
PR8 nonM2MC$^{tom/tom}$
PR8 nonM2MC$^{+/tom}$
Saline M2MC$^{tom/tom}$
Saline M2MC$^{+/tom}$
Saline nonM2MC$^{tom/tom}$
Saline nonM2MC$^{+/tom}$
PR8 M2MC$^{+/tom}$
PR8 nonM2MC$^{+/tom}$
PR8 M2MC$^{tom/tom}$
PR8 nonM2MC$^{tom/tom}$
Saline M2MC$^{+/tom}$
Saline nonM2MC$^{+/tom}$
Saline M2MC$^{tom/tom}$
Saline nonM2MC$^{tom/tom}$
2.0 4.0 6.0 8.0 10.0 12.0 14.0

B

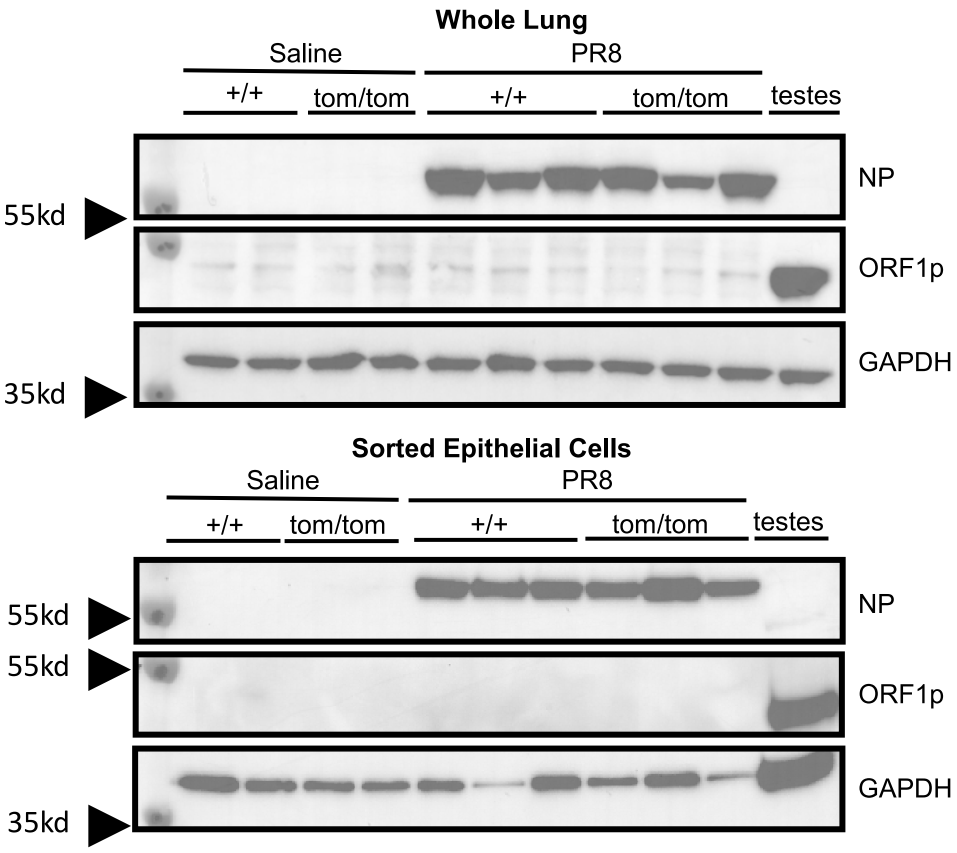
Whole Lung
Saline
PR8
+/+
tom/tom
+/+
tom/tom
testes
55kd
35kd
NP
ORF1p
GAPDH
Sorted Epithelial Cells
Saline
PR8
+/+
tom/tom
+/+
tom/tom
testes
55kd
55kd
35kd
NP
ORF1p
GAPDH

Figure S3

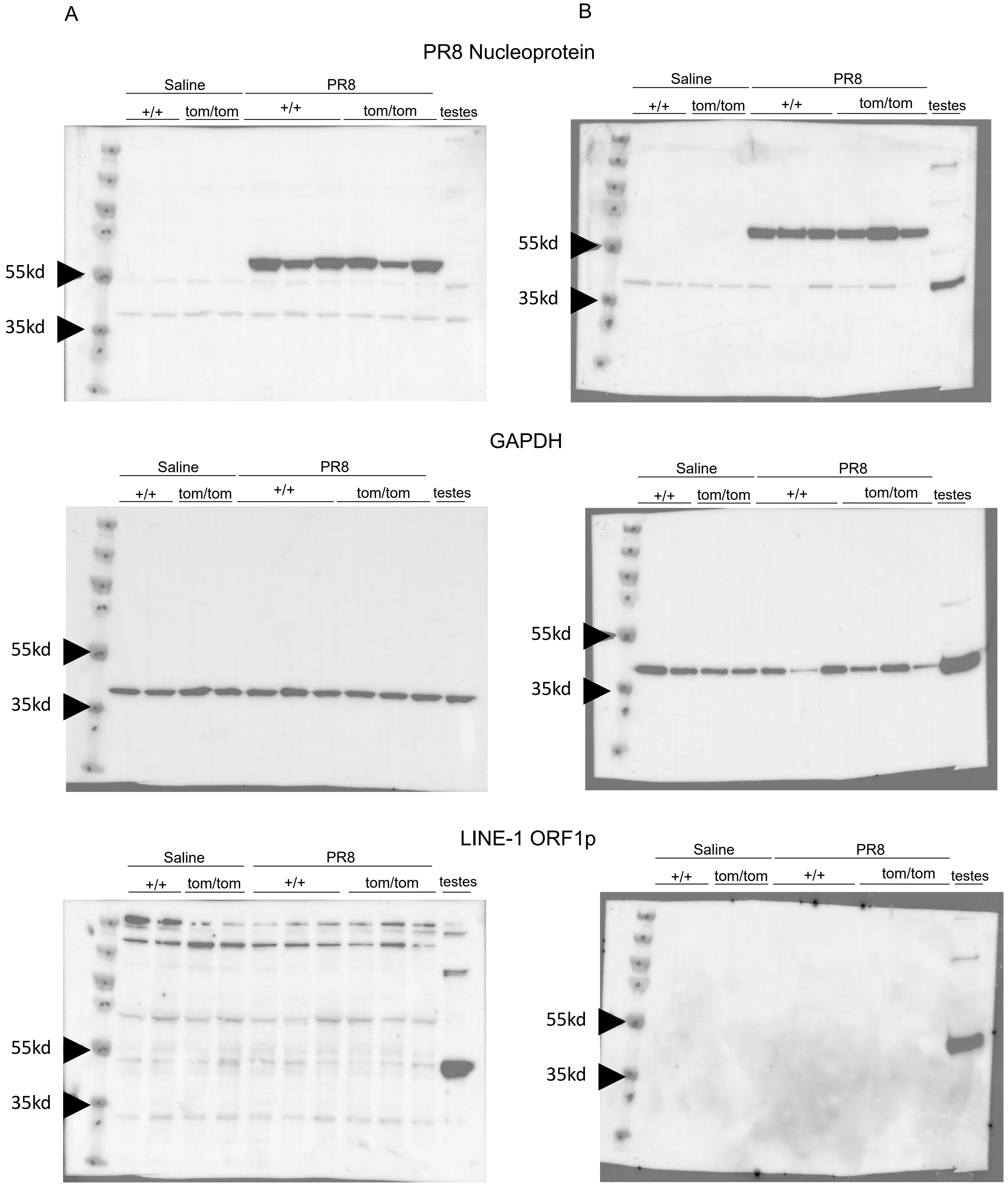
A
B
PR8 Nucleoprotein
Saline
PR8
+/+
tom/tom
+/+
tom/tom
testes
55kd
35kd
Saline
PR8
+/+
tom/tom
+/+
tom/tom
testes
55kd
35kd
GAPDH
Saline
PR8
+/+
tom/tom
+/+
tom/tom
testes
55kd
35kd
Saline
PR8
+/+
tom/tom
+/+
tom/tom
testes
55kd
35kd
LINE-1 ORF1p
Saline
PR8
+/+
tom/tom
+/+
tom/tom
testes
55kd
35kd
Saline
PR8
+/+
tom/tom
+/+
tom/tom
testes
55kd
35kd

Figure S4

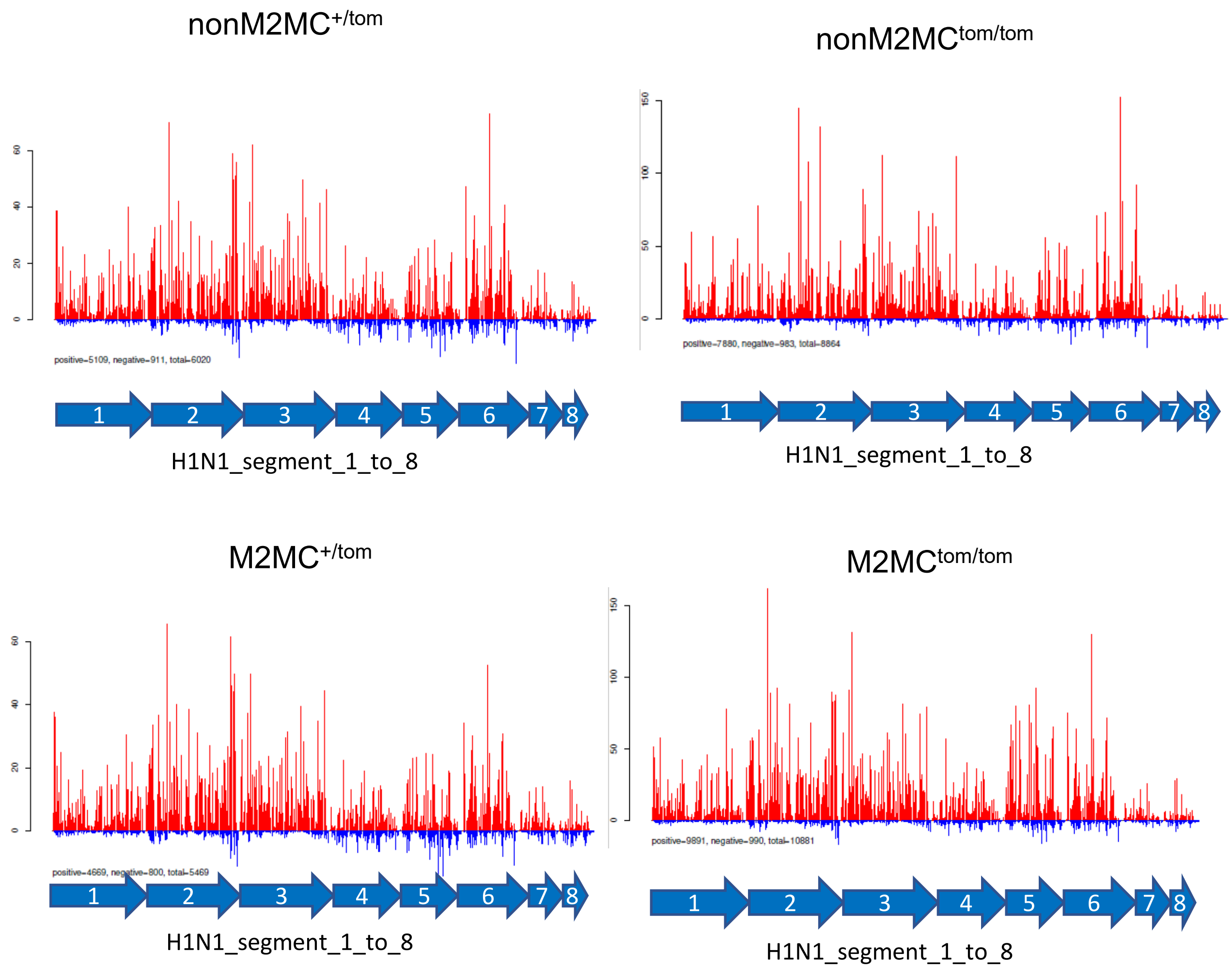
nonM2MC+/tom
0
20
40
60
positive=5109, negative=911, total=6020
1 2 3 4 5 6 7 8
H1N1_segment_1_to_8
nonM2MCtom/tom
0
50
100
150
positive=7880, negative=983, total=8864
1 2 3 4 5 6 7 8
H1N1_segment_1_to_8
M2MC+/tom
0
20
40
60
positive=4669, negative=800, total=5469
1 2 3 4 5 6 7 8
H1N1_segment_1_to_8
M2MCtom/tom
0
50
100
150
positive=9891, negative=990, total=10881
1 2 3 4 5 6 7 8
H1N1_segment_1_to_8

Figure S5

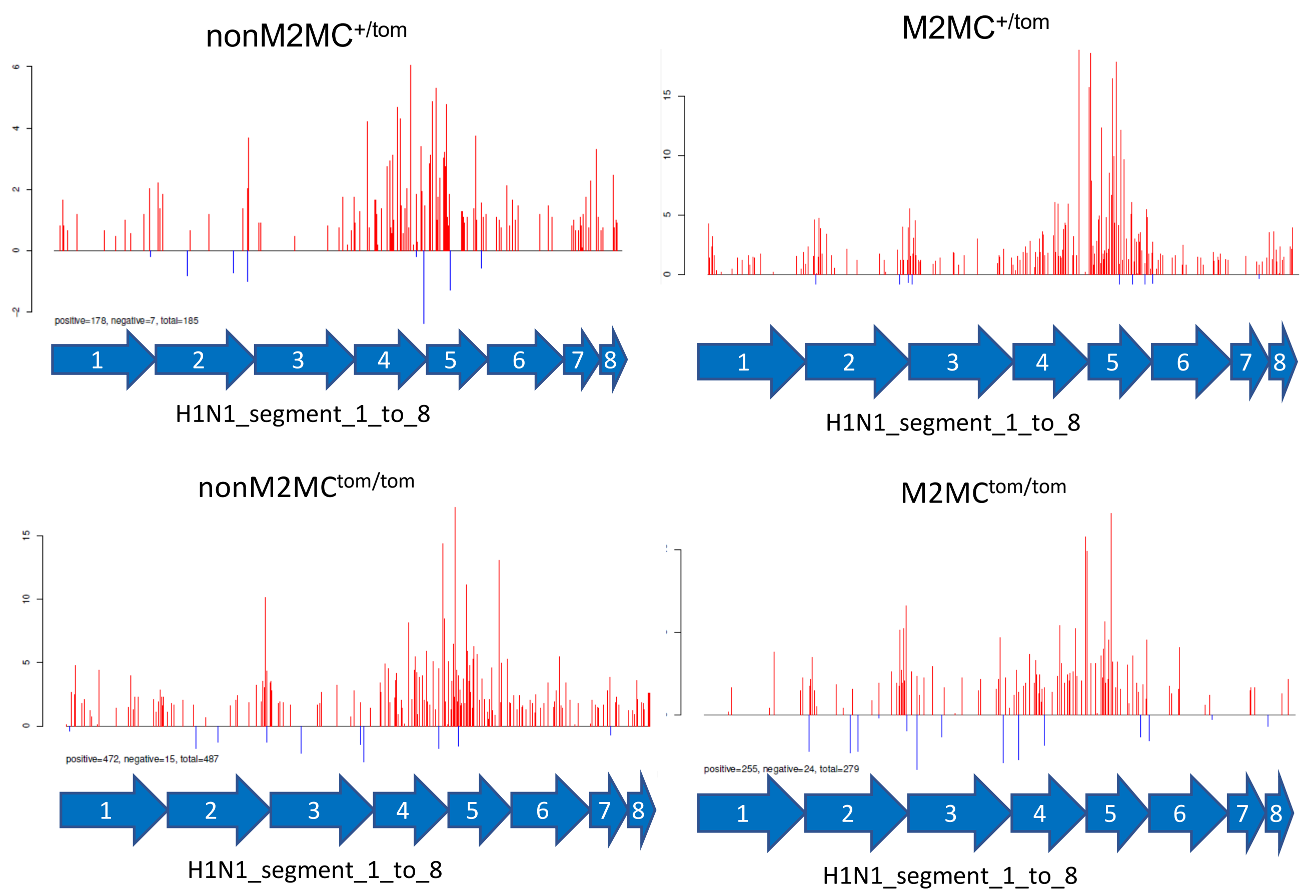
nonM2MC+/tom
positive=178, negative=7, total=185
1
2
3
4
5
6
7
8
H1N1_segment_1_to_8
M2MC+/tom
1
2
3
4
5
6
7
8
H1N1_segment_1_to_8
nonM2MCtom/tom
positive=472, negative=15, total=487
1
2
3
4
5
6
7
8
H1N1_segment_1_to_8
M2MCtom/tom
positive=255, negative=24, total=279
1
2
3
4
5
6
7
8
H1N1_segment_1_to_8

Figure S6

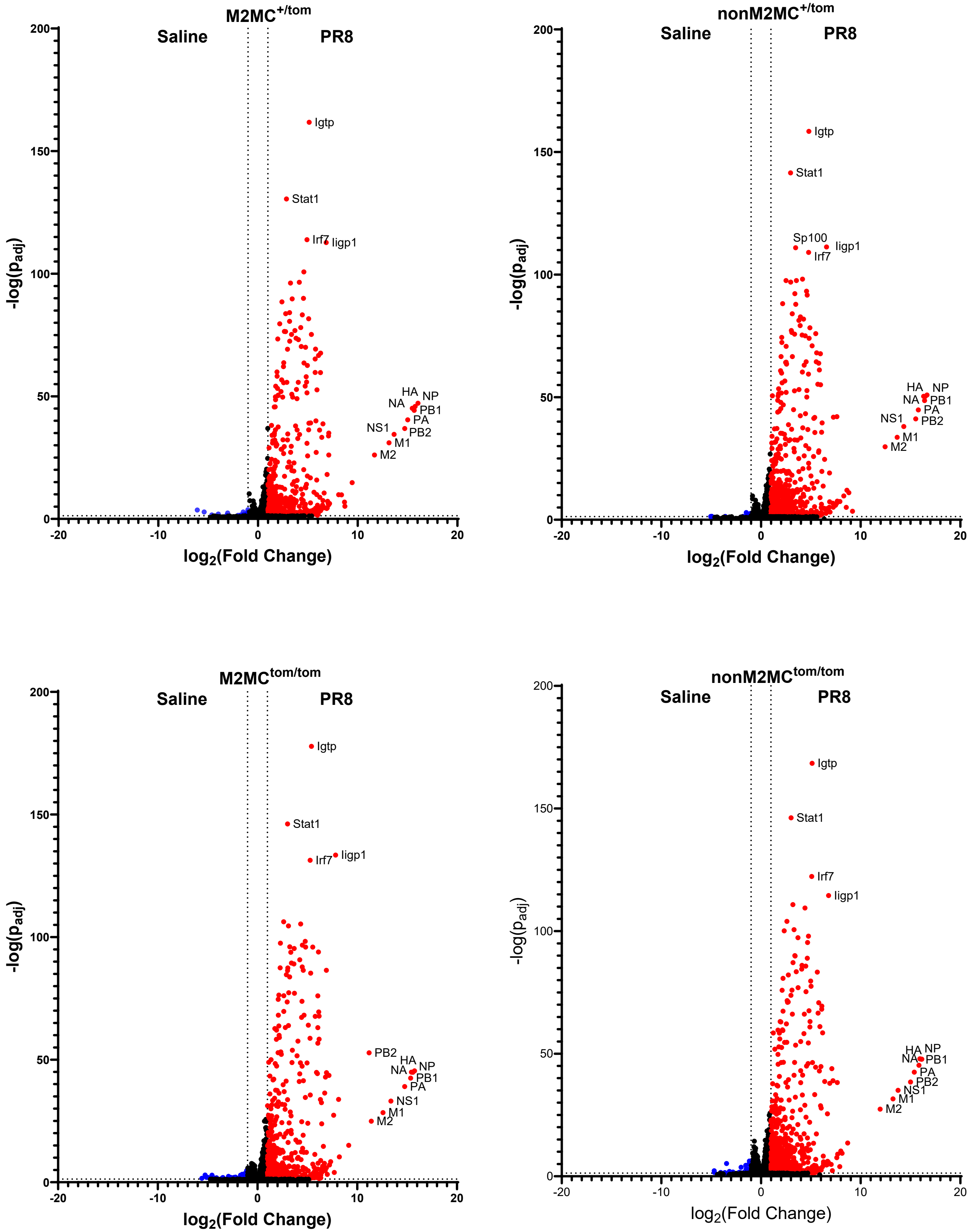
M2MC+/tom
Saline
PR8
Igtp
Stat1
Irf7
Iigp1
HA
NP
NA
PB1
PA
NS1
PB2
M1
M2
-log(padj)
log2(Fold Change)
nonM2MC+/tom
Saline
PR8
Igtp
Stat1
Sp100
Iigp1
Irf7
HA
NP
NA
PB1
PA
NS1
PB2
M1
M2
-log(padj)
log2(Fold Change)
M2MCtom/tom
Saline
PR8
Igtp
Stat1
Irf7
Iigp1
PB2
HA
NP
NA
PB1
PA
NS1
M1
M2
-log(padj)
log2(Fold Change)
nonM2MCtom/tom
Saline
PR8
Igtp
Stat1
Irf7
Iigp1
HA
NP
NA
PB1
PA
PB2
NS1
M1
M2
-log(padj)
log2(Fold Change)

Figure S7

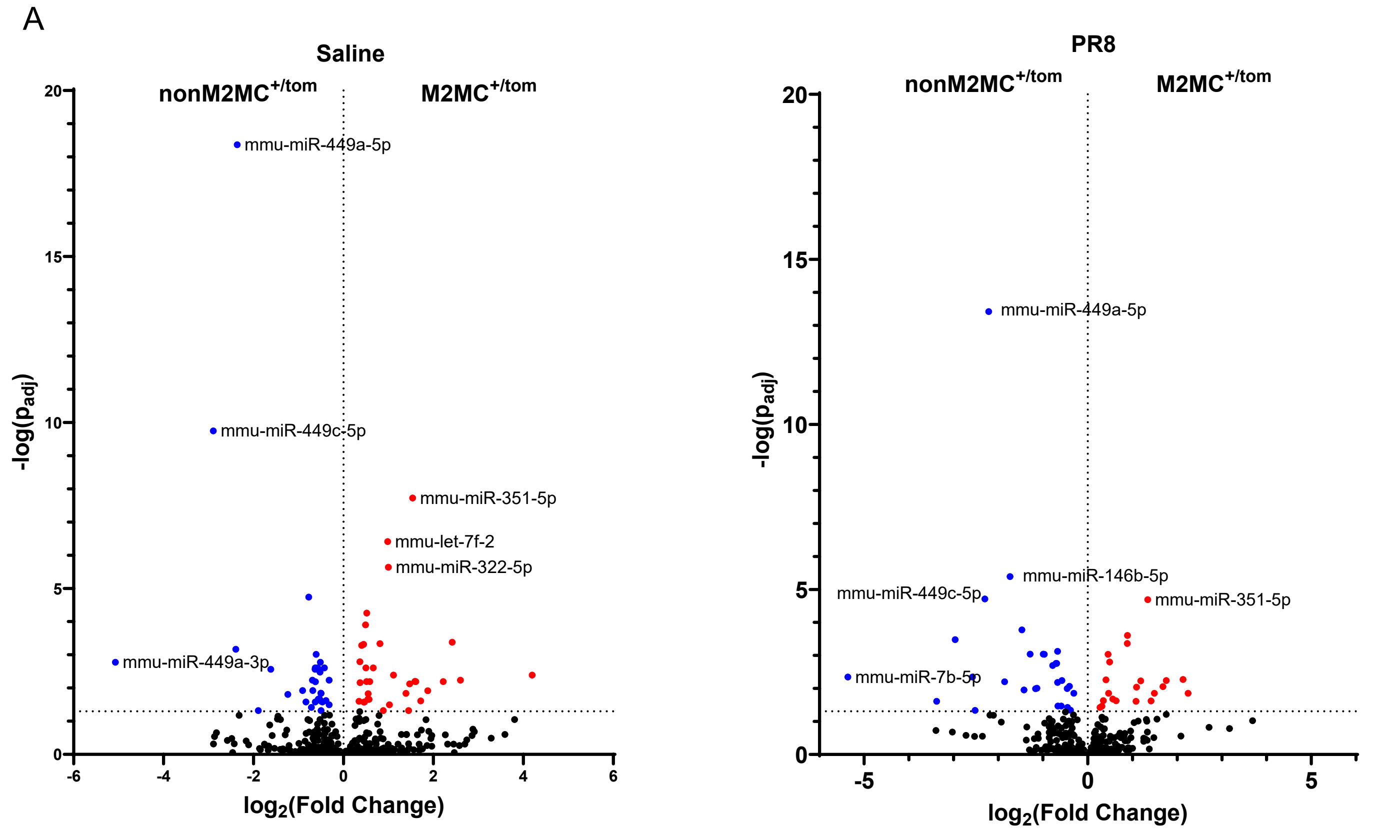

A
Saline
nonM2MC+/tom
M2MC+/tom
mmu-miR-449a-5p
mmu-miR-449c-5p
mmu-miR-351-5p
mmu-let-7f-2
mmu-miR-322-5p
mmu-miR-449a-3p
-log(padj)
log2(Fold Change)
PR8
nonM2MC+/tom
M2MC+/tom
mmu-miR-449a-5p
mmu-miR-146b-5p
mmu-miR-449c-5p
mmu-miR-351-5p
mmu-miR-7b-5p
-log(padj)
log2(Fold Change)

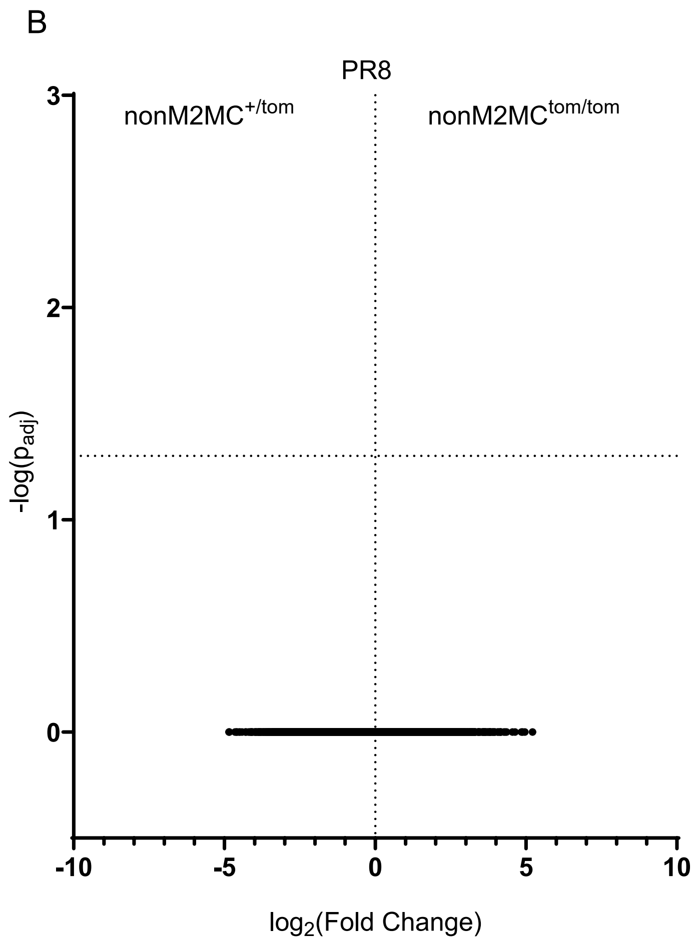

B
PR8
nonM2MC+/tom
nonM2MCtom/tom
-log(padj)
log2(Fold Change)

Figure S8

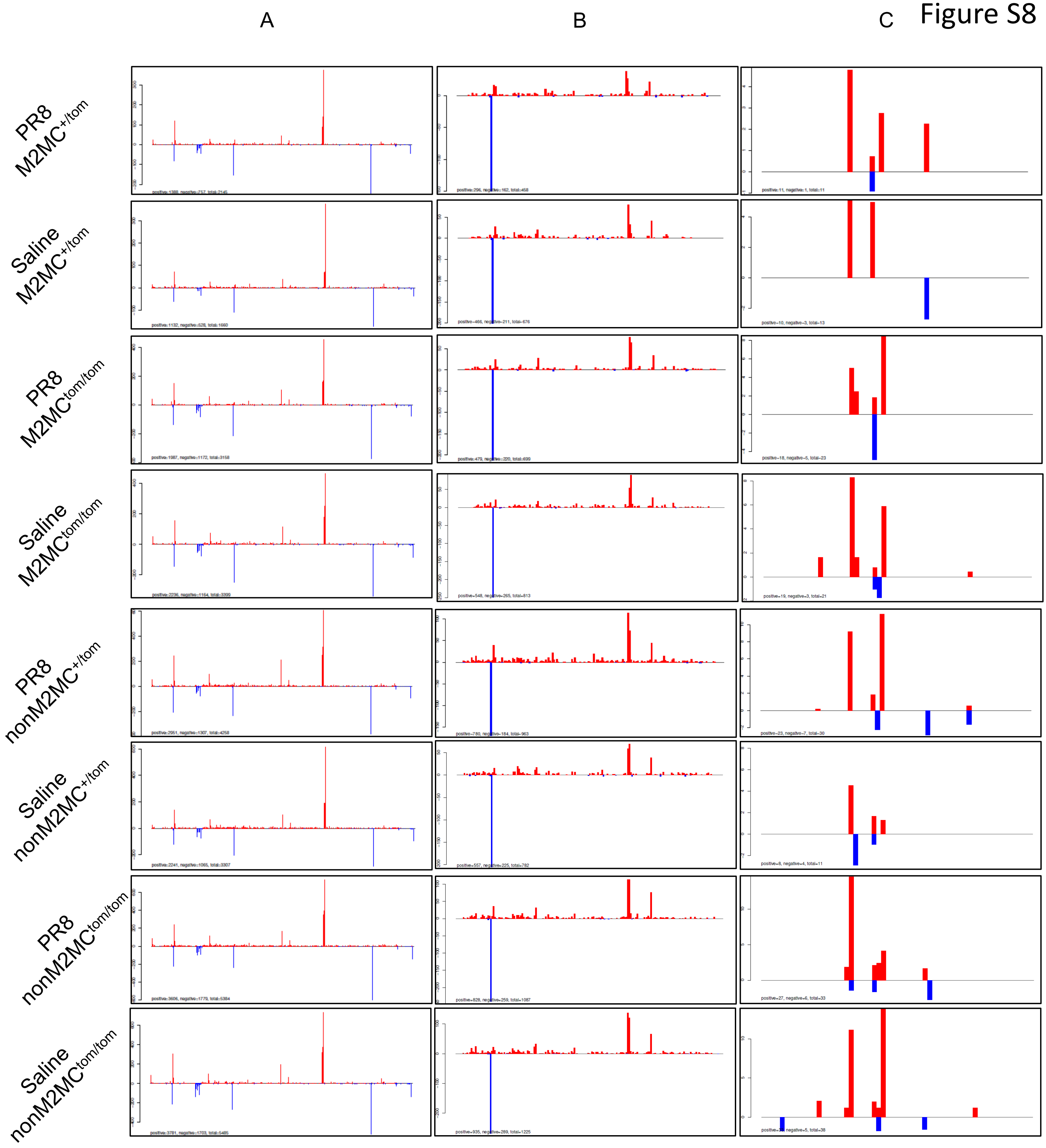
A
B
C
PR8 M2MC+/tom
Saline M2MC+/tom
PR8 M2MCtom/tom
Saline M2MCtom/tom
PR8 nonM2MC+/tom
Saline nonM2MC+/tom
PR8 nonM2MCtom/tom
Saline nonM2MCtom/tom

Figure S9

A

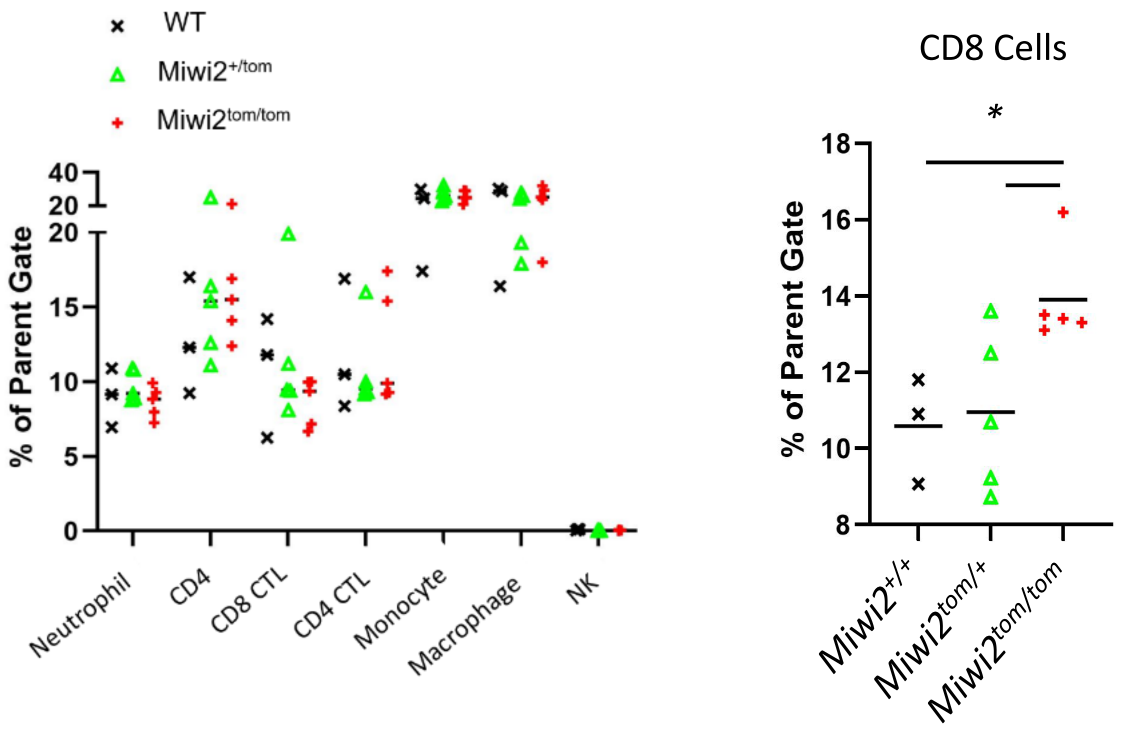
WT
Miwi2+/tom
Miwi2tom/tom
% of Parent Gate
Neutrophil
CD4
CD8 CTL
CD4 CTL
Monocyte
Macrophage
NK
CD8 Cells
*
Miwi2+/+
Miwi2tom/+
Miwi2tom/tom

B

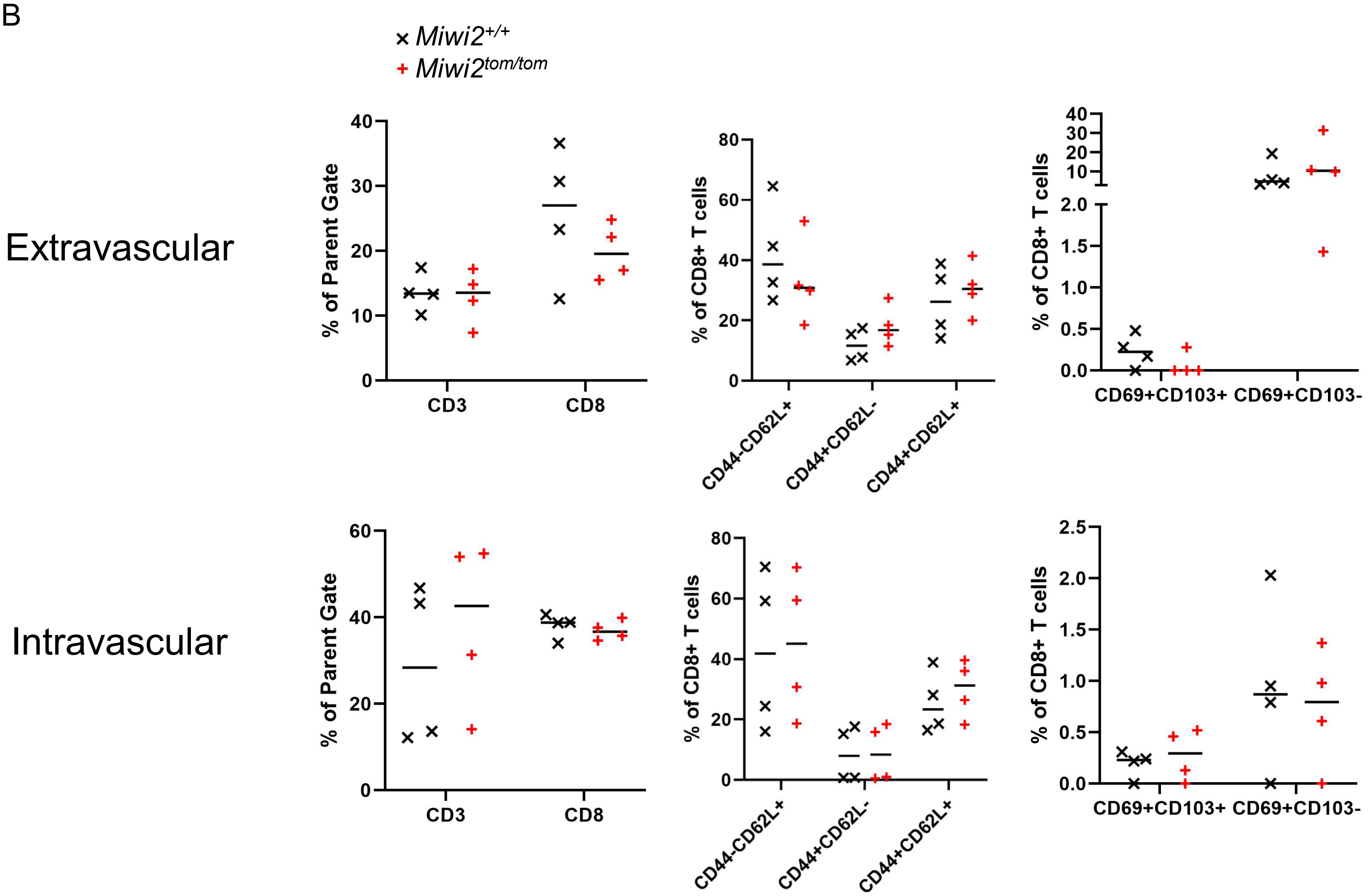
Miwi2+/+
Miwi2tom/tom
Extravascular
Intravascular
% of Parent Gate
% of CD8+ T cells
CD3
CD8
CD44-CD62L+
CD44+CD62L-
CD44+CD62L+
CD69+CD103+
CD69+CD103-

Figure S10

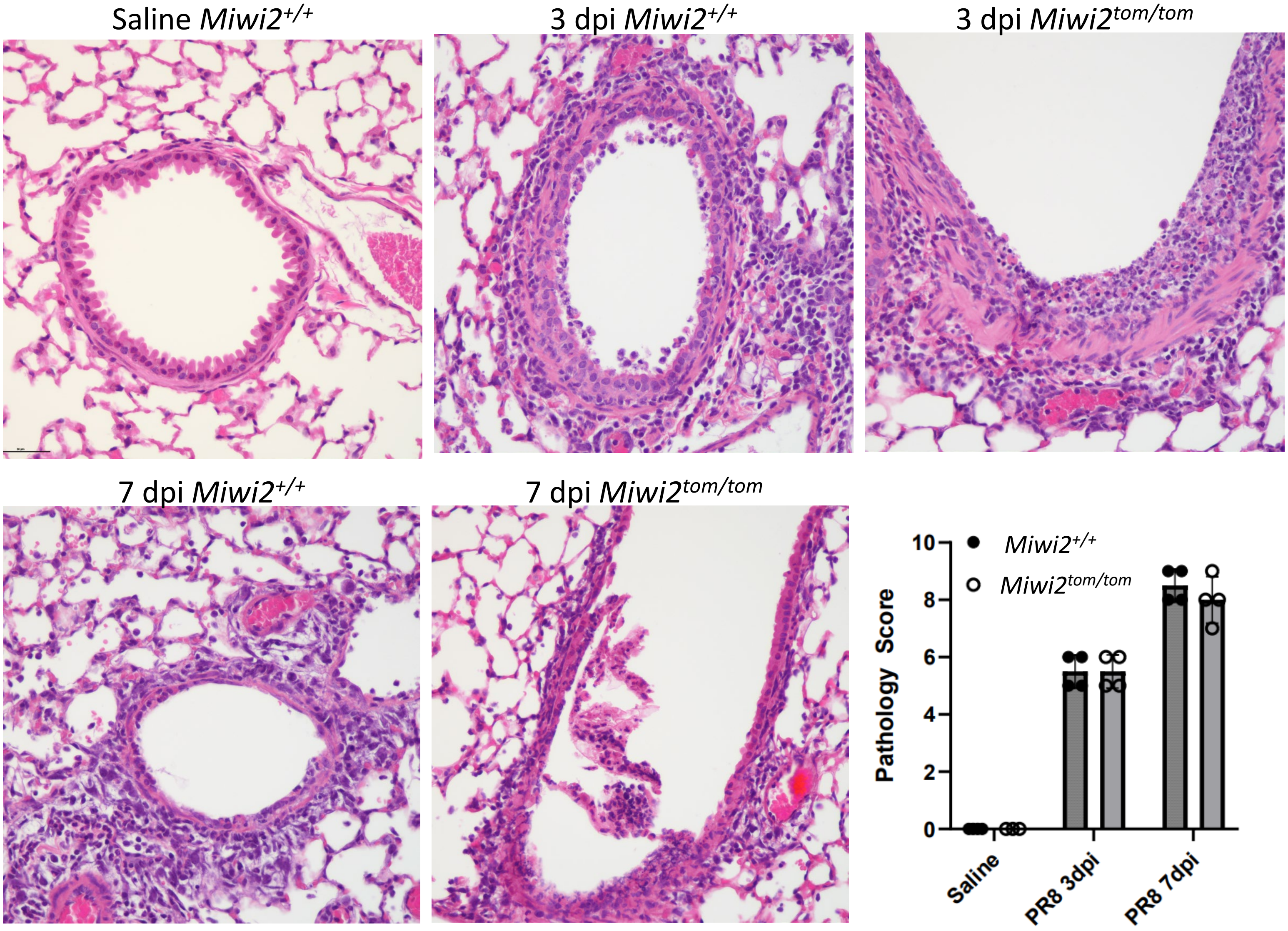
Saline $Miwi2^{+/+}$
3 dpi $Miwi2^{+/+}$
3 dpi $Miwi2^{tom/tom}$
7 dpi $Miwi2^{+/+}$
7 dpi $Miwi2^{tom/tom}$
$Miwi2^{+/+}$
$Miwi2^{tom/tom}$
Pathology Score
0
2
4
6
8
10
Saline
PR8 3dpi
PR8 7dpi

Figure S11

A

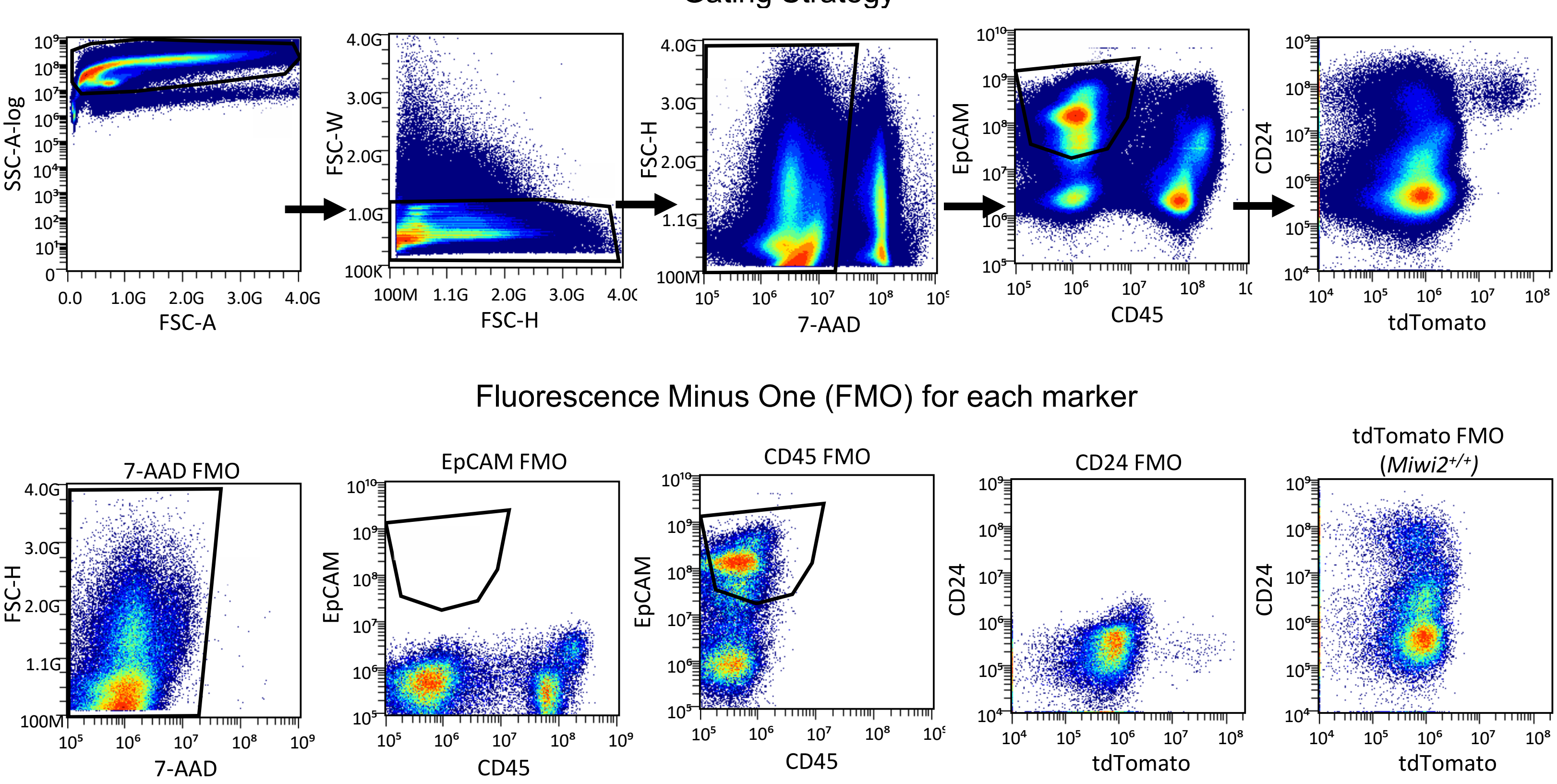

B

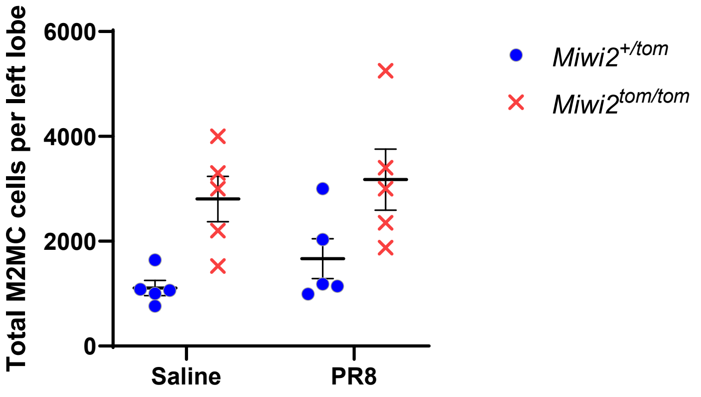

Figure S12

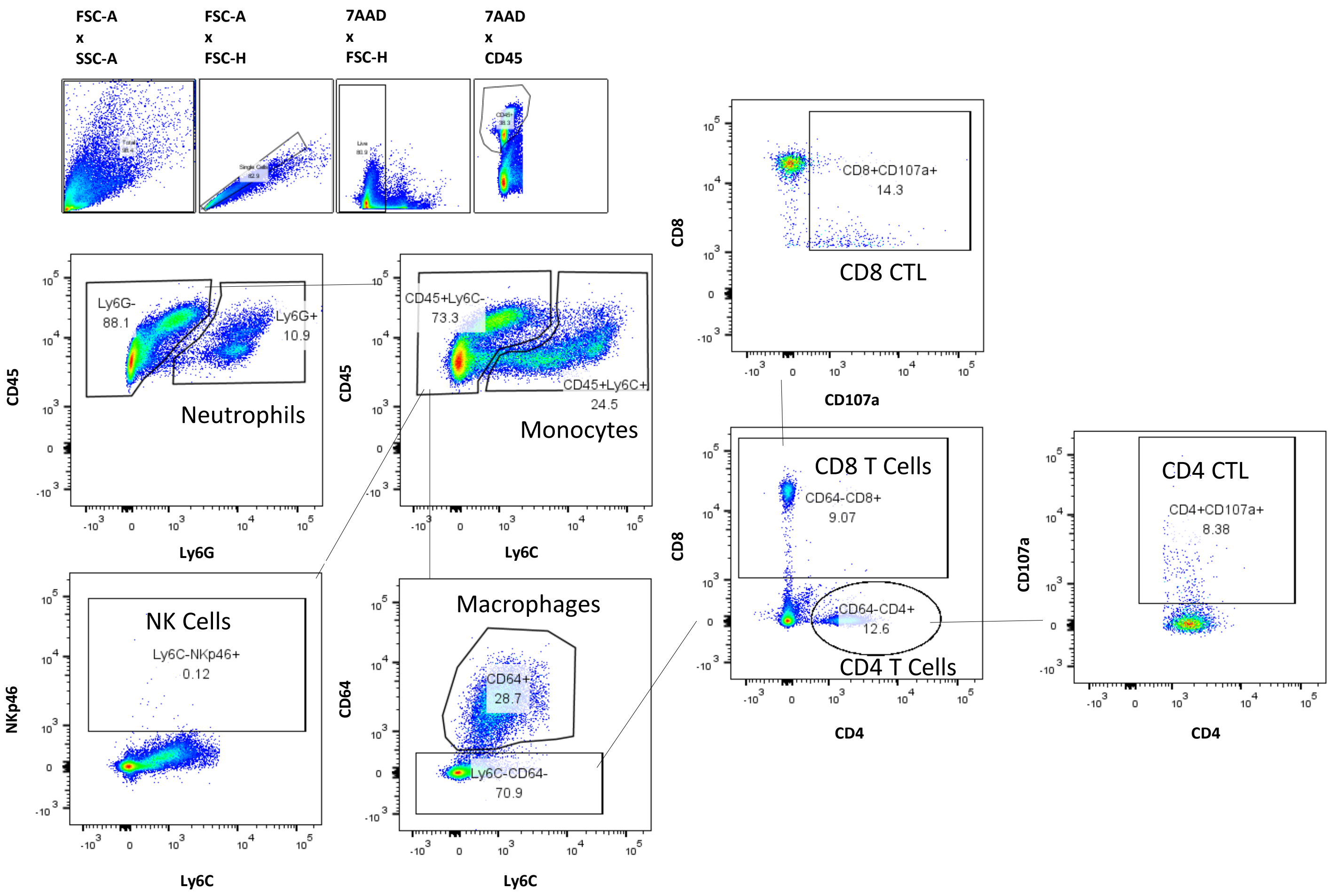

FSC-A
x
SSC-A
FSC-A
x
FSC-H
7AAD
x
FSC-H
7AAD
x
CD45
Ly6G-
88.1
Ly6G+
10.9
Neutrophils
CD45
Ly6G
CD45+Ly6C-
73.3
CD45+Ly6C+
24.5
Monocytes
Ly6C
NK Cells
Ly6C-NKp46+
0.12
NKp46
Ly6C
Macrophages
CD64+
28.7
Ly6C-CD64-
70.9
CD64
CD8+CD107a+
14.3
CD8 CTL
CD8
CD107a
CD8 T Cells
CD64-CD8+
9.07
CD64-CD4+
12.6
CD4 T Cells
CD4
CD4 CTL
CD4+CD107a+
8.38
CD107a

Figure S13

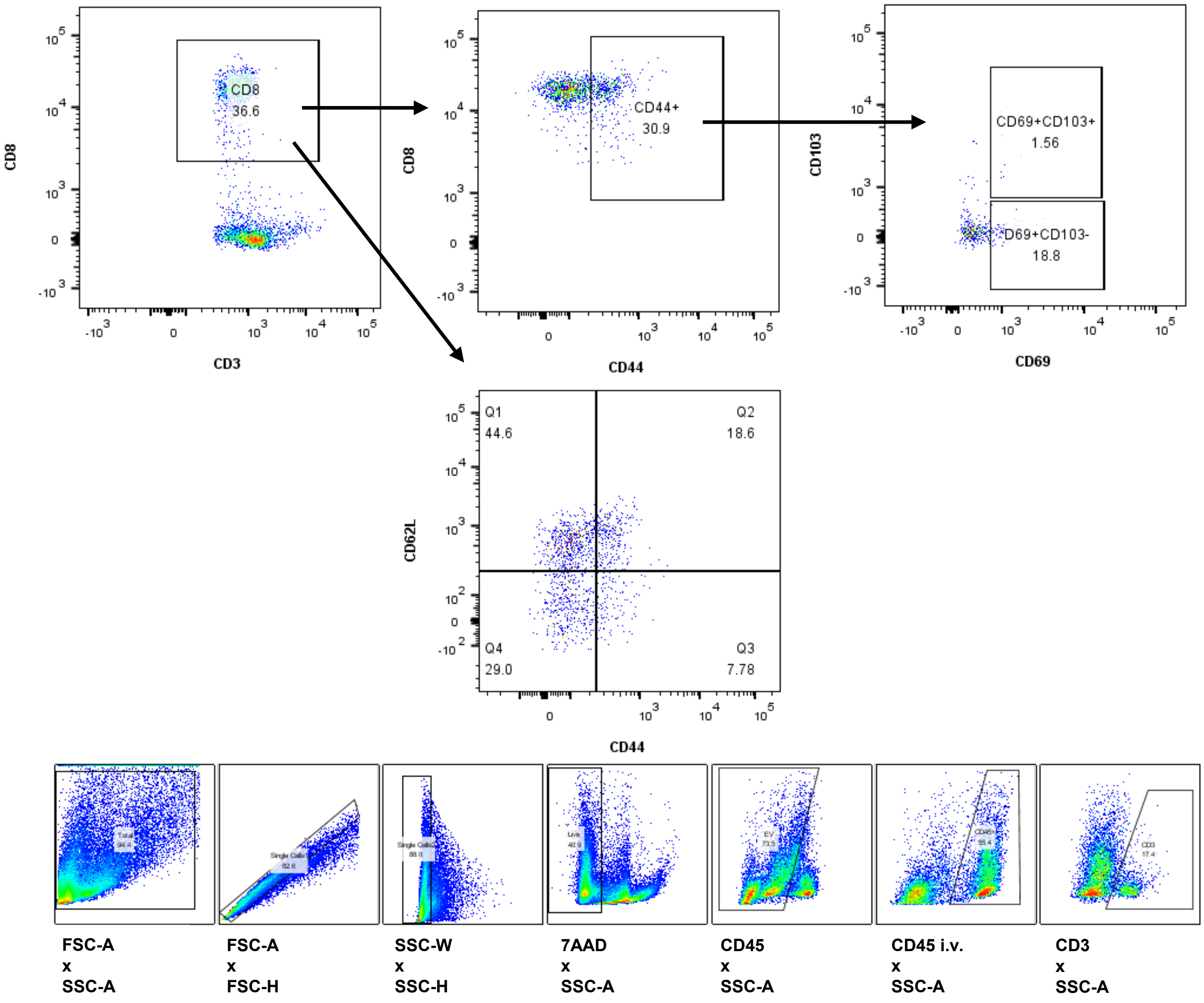

CD8
36.6
CD44+
30.9
CD69+CD103+
1.56
D69+CD103-
18.8
Q1
44.6
Q2
18.6
Q4
29.0
Q3
7.78
CD3
CD8
CD44
CD103
CD69
CD62L
FSC-A x SSC-A
FSC-A x FSC-H
SSC-W x SSC-H
7AAD x SSC-A
CD45 x SSC-A
CD45 i.v. x SSC-A
CD3 x SSC-A

Figure S14

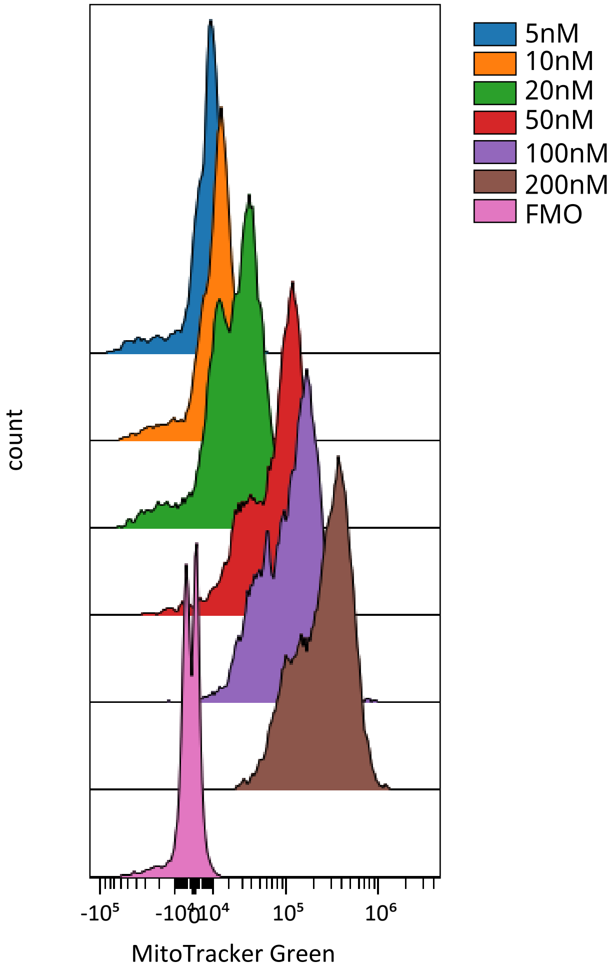

5nM
10nM
20nM
50nM
100nM
200nM
FMO
count
MitoTracker Green

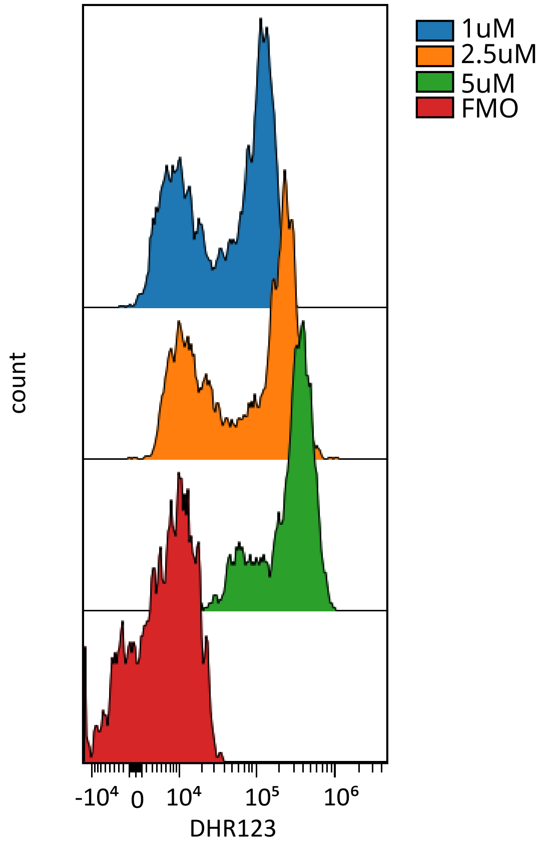

1uM
2.5uM
5uM
FMO
count
DHR123

Supplementary Figure Legends

Figure S1: miRNAs are the predominant species of small RNAs in multiciliated cells.
Length distribution plots of the small RNA libraries in all samples. The majority of small RNAs were miRNAs with a nucleotide (nt) length of 20-24 nts. Two samples had irregular length distributions and were predominantly low in nucleotide length, which likely indicates degraded RNA. These samples were in the PR8 M2MC$^{+/tom}$ and Saline M2MC$^{tom/tom}$ groups (red box) and were excluded in the downstream analysis.

Figure S2: *Miwi2* does not impact steady-state expression of retrotransposons in multiciliated cells.

(A) Genotype (*Miwi2*$^{+/tom}$ versus *Miwi2*$^{tom/tom}$) and cell-type (M2MC versus nonM2MC) RT expression comparisons. (B) Western blot analysis of the LINE-1 open reading frame 1 protein (ORF1p) and PR8 nucleoprotein (NP) in whole lung as well as sorted epithelial cells of intranasally infected mice. Loading confirmed by GAPDH. Testes used as ORF1p positive control.

Figure S3: LINE-1 protein expression is independent of *Miwi2* expression and viral infection in airway epithelial cells
Full gel western blots of LINE-1 ORF1p (~42 kDa), PR8 nucleoprotein (NP, ~63 kDa), and GAPDH (~36 kDa) of *Miwi2*$^{+/+}$ and *Miwi2*$^{tom/tom}$ of whole lung (A) and sorted epithelial cells (C) from Figure S2B.

Figure S4: PR8 long RNA expression pattern is unaltered by *Miwi2* deficiency.
Coverage plots of viral long RNAs in PR8 infected *Miwi2*$^{+/tom}$ and *Miwi2*$^{tom/tom}$ multiciliated cells. All 8 segments of the PR8 genome were concatenated on the x-axis separated by positive (red) and negative (blue) sense RNA.

Figure S5: Distribution of viral small RNAs is unaltered by *Miwi2* deficiency.
Coverage plots of viral small RNAs in PR8 infected *Miwi2*$^{+/tom}$ and *Miwi2*$^{tom/tom}$ multiciliated cells. All 8 segments of the PR8 genome were concatenated on the x-axis separated by positive (red) and negative (blue) sense RNA

Figure S6: PR8 infection induces Stat1 and interferon stimulated gene expression.
Volcano plots of PR8 induced differentially expressed genes of host and viral coding RNA in all samples. (Red, upregulated), (Blue, downregulated), *FDR<0.05.*

Figure S7: M2MC cells are transcriptomically distinct from nonM2MC cells at the miRNA level and *Miwi2* has no impact on the transcriptomic landscape of nonM2MC cells.
(A) Differentially expressed miRNA between M2MC and nonM2MC cells during saline and PR8 treatments. (B) *Miwi2*-dependent differentially expressed genes of PR8 infected nonM2MC cells. *FDR<0.05*.

Figure S8: Small RNAs align to specific sequences on NuMT regions in all multiciliated cell subtypes.
Representative coverage plots of piRNA-like small RNAs that map to the (A) mitochondrial genome region chrM:2494-16207, and to NuMT regions (B) chr1:24611452-24616304, and (C) chr4:80002296-80003747 in all samples. Red and blue peaks represent positive and negative sense RNA, respectively.

Figure S9: *Miwi2* does not affect immune cell recruitment to the lung.
(A) Flow cytometry analysis of immune cell recruitment in 3 dpi lung of $Miwi2^{+/+}$, $Miwi2^{+/tom}$, $Miwi2^{tom/tom}$ mice (Mann-Whitney U test). $^*p<0.05$. (B) A detailed T cell panel was performed to identify changes in CD8 T cell subsets in the extravascular and intravascular regions of the lung.

Figure S10: *Miwi2* deficient lungs do not show significant histopathological differences compared to lungs of wild-type mice at 3 and 7 days post PR8 infection.
Representative images of lung from saline treated $Miwi2^{+/+}$ mice, along with PR8 treated $Miwi2^{+/+}$ and $Miwi2^{tom/tom}$ mice at 3 and 7 dpi, H&E, 200X. Mild to moderate necrotizing bronchiolitis with neutrophilic and mononuclear infiltrates in the bronchiolar mucosa and peribronchiolar regions were consistently observed in all PR8 infected animals. The cumulative pathology scores were comparable between $Miwi2^{+/+}$ and $Miwi2^{tom/tom}$ PR8 infected animals at 3 and 7 dpi.

Figure S11: (A) Gating strategy of sorted multiciliated cells (top). Fluorescence minus one (FMO) plots were generated per each marker to determine positive gates (bottom). (B) Total sorted cell numbers of M2MC cells per left lung lobe

Figure S12: Gating strategy of immune cell panel.

Figure S13: Gating strategy of CD8 T cell panel.

Figure S14: Titration of MitoTracker Green and DHR-123 in $7\text{-AAD}^-\text{CD45}^-\text{EpCAM}^+\text{CD24}^{hi}$ cells with fluorescence minus one (FMO). MFI was examined to determine optimal concentration for staining.

Pathway	Adjust p-value	Genes
Influenza viral RNA transcription and replication	1.00E-05	RPS18;RPL27A;MAGOH;RPL35A;RPL9;RPL39
Cytoplasmic ribosomal proteins	7.18E-05	RPS18;RPL27A;RPL35A;RPL9;RPL39
Influenza infection	1.20E-04	RPS18;RPL27A;RPL35A;RPL9;RPL39
Messenger RNA splicing: major pathway	1.20E-04	HNRNPH1;MAGOH;HNRNPA2B1;U2AF1
Translation	1.20E-04	RPS18;RPL27A;RPL35A;RPL9;RPL39
Gene expression	1.20E-04	RPS18;CBX3;HNRNPH1;RPL27A;MAGOH;HNRNPA2B1;RPL35A;U2AF1;RPL9;RPL39
Cap-dependent translation initiation	1.20E-04	RPL27A;RPL35A;RPL9;RPL39
Respiratory electron transport, ATP biosynthesis by chemiosmotic coupling, and heat production by uncoupling proteins	1.59E-04	NDUFB10;ATP5E;ATP5C1;UQCR10
Electron transport chain	3.93E-04	NDUFB10;ATP5E;ATP5C1;UQCR10
Tricarboxylic acid (TCA) cycle and respiratory electron transport	5.39E-04	NDUFB10;ATP5E;ATP5C1;UQCR10
Parkinson's disease	7.59E-04	NDUFB10;ATP5E;ATP5C1;UQCR10
Oxidative phosphorylation	7.85E-04	NDUFB10;ATP5E;ATP5C1;UQCR10
Capped intron-containing pre-mRNA processing	7.85E-04	HNRNPH1;MAGOH;HNRNPA2B1;U2AF1
Chemiosmotic coupling formation of ATP	0.001405	ATP5E;ATP5C1
Alzheimer's disease	0.001478	NDUFB10;ATP5E;ATP5C1;UQCR10
Huntington's disease	0.001912	NDUFB10;ATP5E;ATP5C1;UQCR10
Messenger RNA processing	0.002605	HNRNPH1;MAGOH;HNRNPA2B1;U2AF1
Protein metabolism	0.006124	RPS18;RPL27A;RPL35A;RPL9;RPL39
Cleavage of growing transcript in the termination region	0.011552	MAGOH;U2AF1
Transport of mature transcript to cytoplasm	0.017232	MAGOH;U2AF1
Transcription	0.017232	CBX3;MAGOH;U2AF1
Disease	0.029979	RPS18;RPL27A;RPL35A;RPL9;RPL39
Androgen receptor signaling, proteolysis, and transcription regulation	0.038285	PRDX1;RNF6
RNA polymerase II transcription	0.047641	MAGOH;U2AF1

Table S1: List of down-regulated pathways and genes associated with *Miwi2* deficiency in M2MC cells.

Gene	Accession Number
Mouse Adapted PR8 Segment 1	CY046986
Mouse Adapted PR8 Segment 2	CY046985
Mouse Adapted PR8 Segment 3	CY046984
Mouse Adapted PR8 Segment 4	CY046979
Mouse Adapted PR8 Segment 5	CY046982
Mouse Adapted PR8 Segment 6	CY046981
Mouse Adapted PR8 Segment 7	CY046980
Mouse Adapted PR8 Segment 8	CY046983
tdtomato	LC311026.1

Table S2: Accession numbers used for annotating the mouse-adapted PR8 genome and tdtomato transcript.
